# Deep Guided Filtering and Detail Recovery-Based Infrared and Visible Image Fusion

**DOI:** 10.3390/s26206510

**Published:** 2026-10-14

**Authors:** Haoran Zhu, Jiaqi Yang, Hangshuo Zhang, Yunqing Liu, Wenying Zhang

**Affiliations:** 1 School of Electronics and Information Engineering, Changchun University of Science and Technology, Changchun 130022, China; 2020800047@cust.edu.cn (H.Z.); 15590165762@163.com (J.Y.); zhs28664@gmail.com (H.Z.); mzlyq@cust.edu.cn (Y.L.); 2 Jilin Engineering Laboratory for Quantum Information Technology, Jilin Engineering Normal University, Changchun 130052, China

**Keywords:** image fusion, deep guided filtering, multi-level decomposition, detail enhancement

## Abstract

To address the issues of poor detail clarity and limited visibility in infrared and visible image fusion under nighttime scenarios, this paper proposes a fusion method based on deep guided filtering and detail recovery. To fully exploit the multiscale features of source images, a multilevel image decomposition model is developed using deep guided filtering and Gaussian filtering, which further decomposes the high-frequency and low-frequency layers obtained by conventional multiscale decomposition into texture, saliency, and base information. In nighttime scenarios, these decomposed components typically suffer from blurring, low contrast, and insufficient brightness, making conventional fusion strategies inadequate for achieving satisfactory results. To cope with these challenges, three fusion strategies specifically tailored for nighttime imaging are devised: a texture information fusion strategy based on an enhancement function, a saliency information fusion strategy leveraging multi-contrast weights, and a base information fusion strategy that integrates the intensity transformation function of infrared images with grayscale difference analysis. To validate the performance of the proposed method, subjective and objective comparative evaluations are conducted on typical datasets including TNO, RoadScene, LLVIP, and MSRS, against classical fusion methods, state-of-the-art approaches, and methods specifically designed for nighttime imaging. Subjectively, the fused images produced by the proposed method exhibit richer and clearer details, along with more prominent thermal targets. Objectively, the proposed method demonstrates consistent advantages across eight metrics: average pixel intensity, standard deviation, average gradient, entropy, spatial frequency, total loss of information measure, perceptual saliency-based metric, and visual information fidelity. Both subjective and objective experimental results confirm that the proposed method can effectively accomplish nighttime image fusion tasks.

## 1. Introduction

Multi-source image data fusion aims to synthesize data acquired from multiple imaging sources, producing a fused image that surpasses any single source in terms of both accuracy and comprehensiveness [1,2]. In various application scenarios, including military surveillance, target tracking, and reconnaissance, the demand for high-fidelity and complete information is critical. Since a single image is often inadequate—either lacking sufficient detail or failing to meet quality standards—data fusion offers an effective means to extract complementary information, thereby increasing the utility of the source data and bolstering the confidence in subsequent decision-making [3,4].

The complementary characteristics of infrared and visible imagery have made their fusion a prominent research branch within the image fusion community [5]. Infrared thermal imaging, which relies on intrinsic radiation differences in the target scene, delivers high detection performance with low sensitivity to illumination and atmospheric disturbances. It also exhibits favorable penetration capability through haze, rain, and snow, enabling all-weather, long-range operation. However, it suffers from poor scene detail rendering and poor alignment with human visual perception. Visible-light sensors, by contrast, capture reflected light from object surfaces to form images that are rich in scene details and consistent with human vision. Yet they are susceptible to adverse weather and other environmental interferences, and they struggle to detect or identify occluded targets [6,7,8]. The fused image obtained by combining the two modalities exhibits more pronounced target regions, finer textural details, and superior overall visual quality.

To date, a plethora of infrared and visible image fusion methodologies have emerged in the literature. Broadly speaking, these approaches can be categorized into two main streams: conventional (traditional) algorithms and deep learning-based paradigms. Among the former group, depending on the core techniques adopted, they are further subdivided into multi-scale transform-based methods, sparse representation-based approaches, and saliency-driven strategies [9].

Within the realm of traditional infrared and visible image fusion, multi-scale transform-based methods stand out as one of the most widely embraced families. Their underlying rationale consists of three sequential steps: first, decomposing the source images into a series of multi-scale representations; second, fusing the transform coefficients according to manually crafted fusion rules; and third, applying an inverse multi-scale transform to reconstruct the final fused image [10]. Early incarnations of multi-scale transforms encompass pyramid decompositions (including Laplacian, contrast, and steerable pyramids), wavelet transforms (such as discrete wavelet transform, dual-tree discrete wavelet transform, and lifting wavelet transform), and multi-scale geometric analysis (exemplified by nonsubsampled contourlet transform and nonsubsampled shearlet transform) [11]. More recently, edge-preserving filter-based approaches have gained popularity within the multi-scale transform category. Such filters are capable of preserving spatial structural consistency while mitigating halo artifacts in the vicinity of sharp edges. Parida et al. [12] first employed an edge-preserving filter in conjunction with a mean filter to extract salient detail features from the source images, then adopted a quantum computing-derived weight map to fuse the detail layers, ultimately yielding a fused image that encapsulates both quantum-level details and salient information. Zhang et al. [13] utilized guided filtering to obtain decomposed components, followed by mean-filter-based weight maps, attention mechanisms, and averaging strategies to fuse the detail, saliency, and base layers, respectively, producing a fused image with elevated contrast. Sparse representation-based methods achieve fusion by constructing over-complete dictionaries, performing sparse coding, and applying specific fusion rules. Li et al. [14] leveraged latent low-rank representation to decompose source images, then employed an averaging strategy and a nuclear norm-based scheme to fuse base and saliency information, respectively. Their fusion outcomes demonstrate excellent preservation of both salient and fine details. Qi et al. [15] utilized a measurement-domain classified sparse dictionary and a dual-channel SPCNN model to fuse the base and detail layers derived from co-occurrence analysis shearlet transform, obtaining results with high contrast and rich textural details. Saliency-based methods, in turn, operate by first capturing prominent target regions within source images to generate saliency maps, from which weight maps are derived to guide the fusion of base and detail layers. Liu et al. [16] extracted visual saliency maps from infrared images to determine fusion weights for the base layer, aiming for enhanced target contrast in the fused output. Chen et al. [17] combined saliency detection with multi-scale decomposition to achieve favorable visual performance while retaining image details. Despite the generally satisfactory results produced by existing traditional fusion algorithms in most scenarios, certain deficiencies persist. For instance, the design of fusion rules has grown increasingly intricate to accommodate complex fusion scenes [18].

In recent years, deep learning has come to dominate the landscape of computer vision, owing to its formidable feature extraction and representation capabilities, and has demonstrated remarkable performance advantages across a spectrum of visual tasks, including image classification, object detection, and semantic segmentation. To further elevate the performance of image fusion algorithms, researchers in this field have extensively explored deep learning-based fusion frameworks, which can be broadly classified into three categories: autoencoder-based, convolutional neural network (CNN)-based, and generative adversarial network (GAN)-based approaches [19]. Autoencoder-based fusion methods are characterized by their tailored design of distinct feature extraction and image reconstruction subnetworks for fusion purposes, coupled with effective training strategies inherent to autoencoder architectures. Li et al. [20] pioneered a method termed DenseFuse for infrared and visible image fusion, which integrates dense block structures into the encoding network. Upon feeding source images into the encoder, CNN layers and dense blocks extract feature maps, which are subsequently merged using an additive strategy and an L1-norm-based scheme. The post-fusion feature maps integrate salient characteristics originating from both modalities. To extract multi-scale features while fully exploiting depth information, Li et al. [21] proposed an architecture built upon the Nest network and spatial/channel attention mechanisms. By computing attention via the L1 norm, the Nest-based framework preserves substantial information from the input data from a multi-scale perspective. Although autoencoder-based fusion algorithms have achieved encouraging results with notable performance improvements, they still rely on manually specified operations within the fusion framework—namely, the fusion strategies themselves. CNN-based fusion algorithms typically harness the formidable fitting capacity of neural networks to extract and reconstruct effective information under the guidance of meticulously designed loss functions or ground-truth labels. Liu et al. [22] presented the first CNN-based approach for infrared and visible image fusion. Their method employs a Siamese CNN to derive weight maps that reflect pixel-level activity information from both modalities. Considering the distinct imaging mechanisms of infrared and visible sensors, this approach utilizes image pyramids to perform fusion at multiple scales, with decomposition coefficients automatically adjusted via a local-similarity-based strategy. Liu et al. [23] later introduced an architecture-search-based image fusion method capable of adaptively constructing efficient and effective feature extraction, feature fusion, and image reconstruction networks tailored to different fusion tasks. While CNN-based methods have adopted certain alternatives to ground-truth labels—substantially enhancing fused image quality—the absence of standard ground-truth references continues to constrain the full realization of CNN’s potential in the infrared and visible image fusion domain. GAN-based fusion algorithms leverage a discriminator to compel the generator’s output to approximate the target distribution in terms of probability, thereby implicitly accomplishing feature extraction, fusion, and image reconstruction. Ma et al. [24] pioneered the introduction of GANs into infrared and visible image fusion, proposing FusionGAN. During the adversarial process, the generator progressively fits the distribution of gradient information in the fused image to that of the visible image, while concurrently preserving infrared intensity information. When the discriminator is no longer able to distinguish between the fused image and the visible image, it is considered that the fused image has adequately preserved the fine details from the visible modality—meaning that the fusion result exhibits both distinct, prominent targets and abundant textures. Overall, GAN-based methods tend to produce fusion outcomes that align well with expectations; however, maintaining equilibrium between the generator and discriminator during training remains a nontrivial challenge, warranting further investigation in this promising direction.

In summary, although infrared and visible image fusion technology has achieved considerable progress, there still remain many issues that urgently need to be addressed by researchers. First, most image fusion algorithms are designed based on daytime scenes, assuming that the details in the fused image are primarily provided by the visible image. However, in practical applications, infrared and visible image fusion often needs to be performed under nighttime conditions, where the visible image is captured in low-light environments and its detailed information is typically blurred or submerged in darkness. Existing fusion methods can only rely on the infrared image to provide limited detail features for the fused result, which obviously cannot meet the demand for rich scene details required by the infrared and visible image fusion task. Second, some fusion methods adopt a strategy of first enhancing the visible image and then performing fusion to improve the brightness, contrast, and clarity of the fused result. However, performing image enhancement and image fusion independently tends to cause incompatibility between the two processes, ultimately producing unsatisfactory fused images with noticeable distortions, e.g., color or structural distortion. Moreover, aside from direct fusion, alternative low-light paradigms can also address nighttime vision tasks. Fernando et al. [25], for instance, used a modified CycleGAN to develop a night-to-day image translation model. This model converts RGB and infrared (IR) data into daytime-like representations, thereby enhancing holistic perception and object detection under low-light conditions. Nevertheless, such approaches fail to fully leverage cross-modal complementary information. To address the above issues, this paper proposes a novel fusion framework by combining deep guided filtering and detail recovery. The main improvements of the proposed algorithm are as follows:

(1) A deep guided filtering-based multilevel image decomposition model is proposed, which further decomposes the high-frequency and low-frequency layers derived from conventional multi-scale decomposition into texture, saliency, and base information. The main contribution of this decomposition model lies in its capacity to thoroughly exploit the multi-scale feature information inherent in both infrared and visible images.
(2) A detail enhancement-oriented fusion rule is developed for the integration of texture information. By leveraging the weighted sum of an improved Laplacian operator, this strategy is built upon an enhancement function specifically intended to sharpen blurred details within the fused output.
(3) A contrast-aware fusion scheme is devised for the integration of saliency information. This method constructs multi-contrast fusion weights using foreground-background contrast features extracted by the local standard deviation filter and the local range filter, with the goal of preserving salient scene and target details from both infrared and visible sources.
(4) A base information fusion strategy is introduced, which relies on the intensity transformation function of infrared images and grayscale difference between modalities. The key novelty of this contribution is twofold: first, the intensity transformation function is employed to elevate the global luminance of the fused image, thereby rendering details in dark regions visible; second, a grayscale-difference-based fusion rule is adopted to enhance the contrast of infrared targets against the fused background.

## 2. Proposed Fusion Method

**Figure 1:**
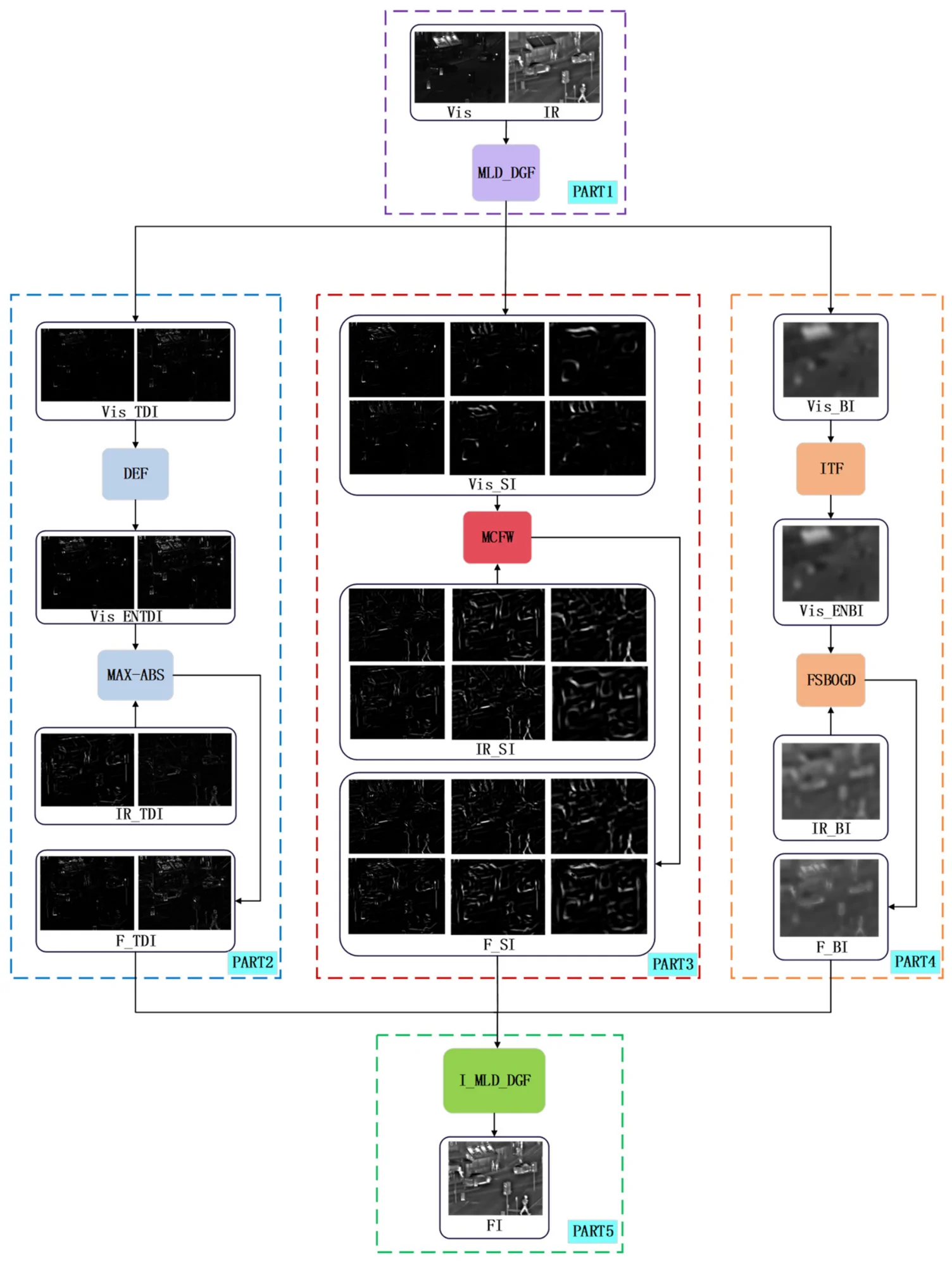
The framework of the proposed method. **Part 1** is used for multilevel decomposition. **Part 2** is used for fusing texture information. **Part 3** is used for fusing saliency information. **Part 4** is used for fusing base information. **Part 5** is used for reconstructing the fused image. **MLD_DGF** represent deep guided filtering-based multilevel decomposition. **I_MLD_DGF** represent inverse operation of deep guided filtering-based multilevel decomposition. **Vis_TDI** represent visible texture detail image. **DEF** represent detail enhancement function. **Vis_ENTDI** represent enhanced visible texture detail image. **MAX-ABS** represent the “maximum absolute value” strategy. **IR_TDI** represent infrared texture detail image. **F_TDI** represent fused texture detail image. **Vis_SI** represent visible saliency image. **MCFW** represent multi-contrast fusion weights. **IR_SI** represent infrared saliency image. **F_SI** represent fused saliency image. **Vis_BI** represent visible base image. **ITF** represent intensity transformation function. **Vis_ENBI** represent enhanced base image. **FSBOGD** represent fusion strategy based on grayscale difference. **IR_BI** represent infrared base image. **F_BI** represent fused base image. **FI** represent fused image.

The architecture of the proposed fusion framework is illustrated in Figure 1, which comprises five sequential modules. In part 1, a deep guided filtering-based multilevel image decomposition model is employed to disentangle the original infrared and visible images into three distinct components, namely texture information, saliency information (strong edges), and base information. In part 2, the visible texture information is first adaptively enhanced via an enhancement function derived from the weighted sum of an improved Laplacian operator, followed by fusion of the enhanced visible texture and the infrared texture using an absolute-value-maximization rule to yield the fused texture component. In part 3, the foreground contrast features are extracted from the infrared saliency information using a local range filter, while the background contrast features are simultaneously extracted from the visible saliency information via a local standard deviation filter. These extracted features are then exploited to construct multi-contrast fusion weights, through which the fused saliency component is obtained. In part 4, the visible base information is first enhanced by applying the intensity transformation function of infrared images, and then the fused base component is derived using a grayscale-difference-based fusion rule. Finally, in part 5, the ultimate fused image is reconstructed by integrating all the fused decomposed components.

### 2.1. Deep Guided Image Filtering

Section 2.1 corresponds to Parts 1 and 5 of the fusion framework shown in Figure 1. In general, an image encompasses features spanning multiple scales; nevertheless, for a given image processing task, only a subset of key scale features needs to be retained [26]. Multilevel image decomposition is capable of accurately disentangling features at different scales. When applied to infrared and visible image fusion, it not only mitigates the adverse effects of insignificant scale features on algorithmic performance but also enhances computational efficiency [27]. Edge-preserving filters, which maintain structural consistency while suppressing halo artifacts in the vicinity of edges, have garnered considerable favor among researchers in the field of image decomposition. Among the plethora of multilevel decomposition approaches built upon edge-preserving filters, guided filtering stands out as one of the most prevalent [28]. For any local window centered at pixel k (e.g., a square window of radius r), guided filtering assumes that the output image O and the guidance image G adhere to the following linear relationship:

(1) $$O_{i}\;=\;a_{k}G_{i}+b_{k}\;\forall i\in \omega_{k}$$

Here, the linear coefficients $a_{k}$ and $b_{k}$ are treated as constants computed within each local window $\omega_{k}$. To ensure that the output image both resembles the guidance image and remains faithful to the input image I, guided filtering achieves this objective by minimizing the following cost function within the window:

(2) $$L(a_{k},b_{k})=\sum_{i\in \omega_{k}}\left((a_{k}G_{i}+b_{k}-I_{i}{)}^{2}+\varepsilon_{1}a_{k}^{2}\right)$$

Here, $\varepsilon_{1}$ is a tunable parameter that prevents $a_{k}$ from taking excessively large values. The cost function, denoted as $L(a_{k},b_{k})$, is formulated as a classical linear regression problem. By taking the partial derivatives of $a_{k}$ and $b_{k}$ and setting them to zero, the optimal $a_{k}$ and $b_{k}$ within the window $\omega_{k}$ can be solved.

**Figure 2:**
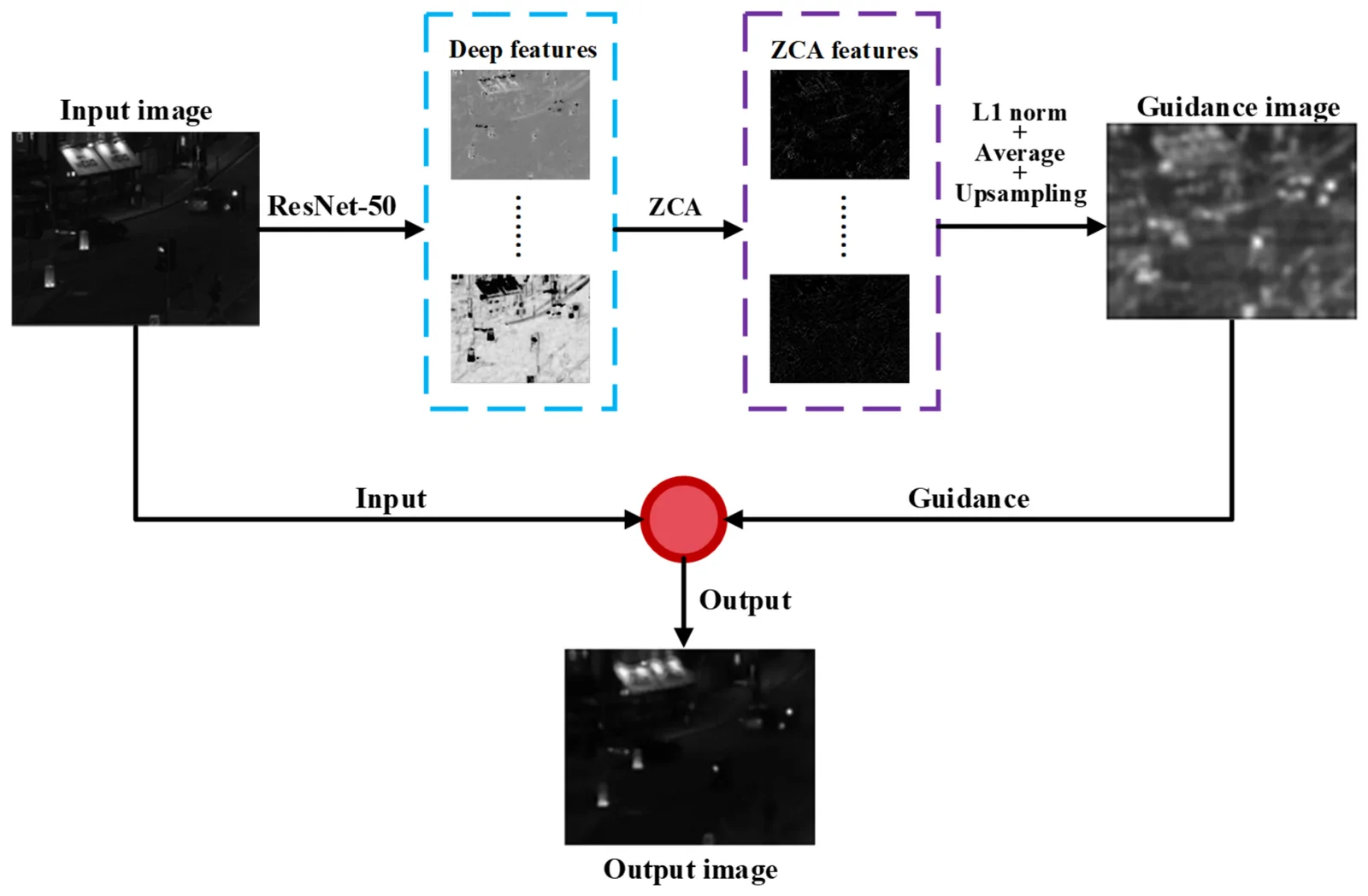
Flowchart of deep guided filtering.

According to the operational principle of guided filtering, this technique effectively transfers structural information—such as edges and textures—from the guidance image into the output image. In other words, the resulting output exhibits edges and textures that closely resemble those of the guidance image. Conventional guided filtering typically employs the original image itself as the guidance image. However, since the original image contains a substantial amount of irrelevant or redundant information, the detail layers produced during multilevel decomposition tend to struggle in capturing finer subtleties within edge and texture regions, which in turn compromises the discriminability of targets and the interpretability of scenes in the fused outcome. To address this limitation, we propose a deep guided filtering strategy, the flowchart of which is depicted in Figure 2. The proposed deep guided filtering pipeline comprises two principal steps: guidance image extraction and image smoothing.

The most critical step in deep guided filtering is the acquisition of the guidance image. As analyzed above, the guidance image for guided filtering should ideally exclude all extraneous interference and retain only salient features, thereby enabling the effective smoothing of finer details. Deep learning techniques, renowned for their superior feature representation capabilities, are capable of extracting deep features rich in salient information from raw images [29]. In theory, training a fusion network demands a substantial volume of training data. However, infrared and visible images, as typical multimodal data, often suffer from limited training samples due to the heavy burden of image registration and the scarcity of ground-truth labels (i.e., ideal fused images). This limitation falls far short of the requirements for holistic network training. Fortunately, deep features extracted from pre-trained large-scale neural networks exhibit strong generalizability, making it feasible to leverage abundant deep features for image fusion tasks even in the absence of large training datasets. The residual network-based convolutional neural network (ResNet), pre-trained on ImageNet, elegantly addresses the degradation and vanishing gradient problems in deep networks through the incorporation of simple skip-connection structures [30]. Among the various ResNet variants, ResNet-50 strikes a favorable balance between sufficient depth for robust feature extraction and computational complexity that remains manageable for most research and applications. Consequently, ResNet-50 is selected as the feature extractor for constructing the deep guidance image in this work. ResNet-50 comprises five convolutional blocks from shallow to deep layers, namely conv1, conv2, conv3, conv4, and conv5. Deeper convolutional blocks yield more salient features but at the cost of increased spatial information loss, which may result in blurred edges and inaccurate localization in the output image. Therefore, the deep features captured by conv4 (with a maximum output channel number of 1024) are employed to construct the guidance image, and the resulting feature maps are denoted as $G_{K}^{4,1:1024}$. As can be observed from the deep features extracted in Figure 2, the numerical ranges of different feature maps vary considerably—with white representing higher values and black indicating lower ones. Such discrepancies may render certain feature maps ineffective during guidance image construction. To address this issue, zero-phase component analysis (ZCA) is adopted for further processing of the raw deep features. ZCA is a specialized whitening method that operates through a three-step procedure—rotation, scaling, and inverse rotation—which eliminates data correlation and normalizes variance while maximally preserving the global structure of the data, ensuring that the transformed results remain as similar as possible to the input data in the original feature space. The formulation of ZCA applied to the deep features is given as follows:

(3) $$\begin{array}{l} {\hat{G}}_{K}^{4;j}=s_{K}^{4;j}\times G_{K}^{4;j},\;s_{K}^{4;j}=U{\left(\sum +\varepsilon_{2}I\right)}^{-\frac{1}{2}}U^{T}\\ s.t.Co_{K}^{4;j}=G_{K}^{4;j}\times (G_{K}^{4;j})T,\;Co_{K}^{4;j}=U\Sigma V^{T} \end{array}$$

In this formulation, $G_{K}^{4;j}$ denotes the j-th deep feature map extracted from the convolutional block conv4, while ${\hat{G}}_{K}^{4;j}$ represents its corresponding ZCA-transformed counterpart. The matrix $Co_{K}^{4;j}$ stands for the covariance matrix of the j-th deep feature map from conv4. Here, $j\in \left\{1,2,\cdots ,1024\right\}$, and I denotes the identity matrix. The matrices U, Σ, and $V^{T}$ are the left singular vector matrix, the singular value matrix, and the transpose of the right singular vector matrix, respectively, all obtained via singular value decomposition (SVD) of $Co_{K}^{4;j}$. The term $\varepsilon_{2}$ is a very small constant introduced primarily to ensure the invertibility of the matrix. The subscript $K\in \left\{Vis,IR\right\}$ denotes the visible and infrared images. As can be intuitively observed from the ZCA-transformed features in Figure 2, the ZCA operation effectively enhances the salient features within the depth map.

The ZCA operation imposes a sparsity constraint on the deep features, rendering the ZCA-transformed features ${\hat{G}}_{K}^{4,1:1024}$ more sparse along the channel dimension. Consequently, we first employ the L1 norm in conjunction with a patch-based averaging operation centered at ${\hat{G}}_{K}^{4,1:1024}$ with a radius of t=2 to construct an initial guidance image. The patch-based averaging operation is adopted to render the guidance image more natural and visually coherent. Meanwhile, since the features from conv4 have undergone multiple pooling operations, they need to be resized back to the original image dimensions. To this end, bilinear interpolation is subsequently applied to perform upsampling, yielding the final guidance image $G_{K}^{4,*}$. The formulations corresponding to the above procedures are given as follows:

(4) $$G_{K}^{4,*}=\frac{\sum_{p=x-t}^{x+t}\sum_{q=y-t}^{y+t}{\left\|{\hat{G}}_{K}^{4,1:1024}(p,q)\right\|}_{1}}{{\left(2t+1\right)}^{2}}$$

**Figure 3:**
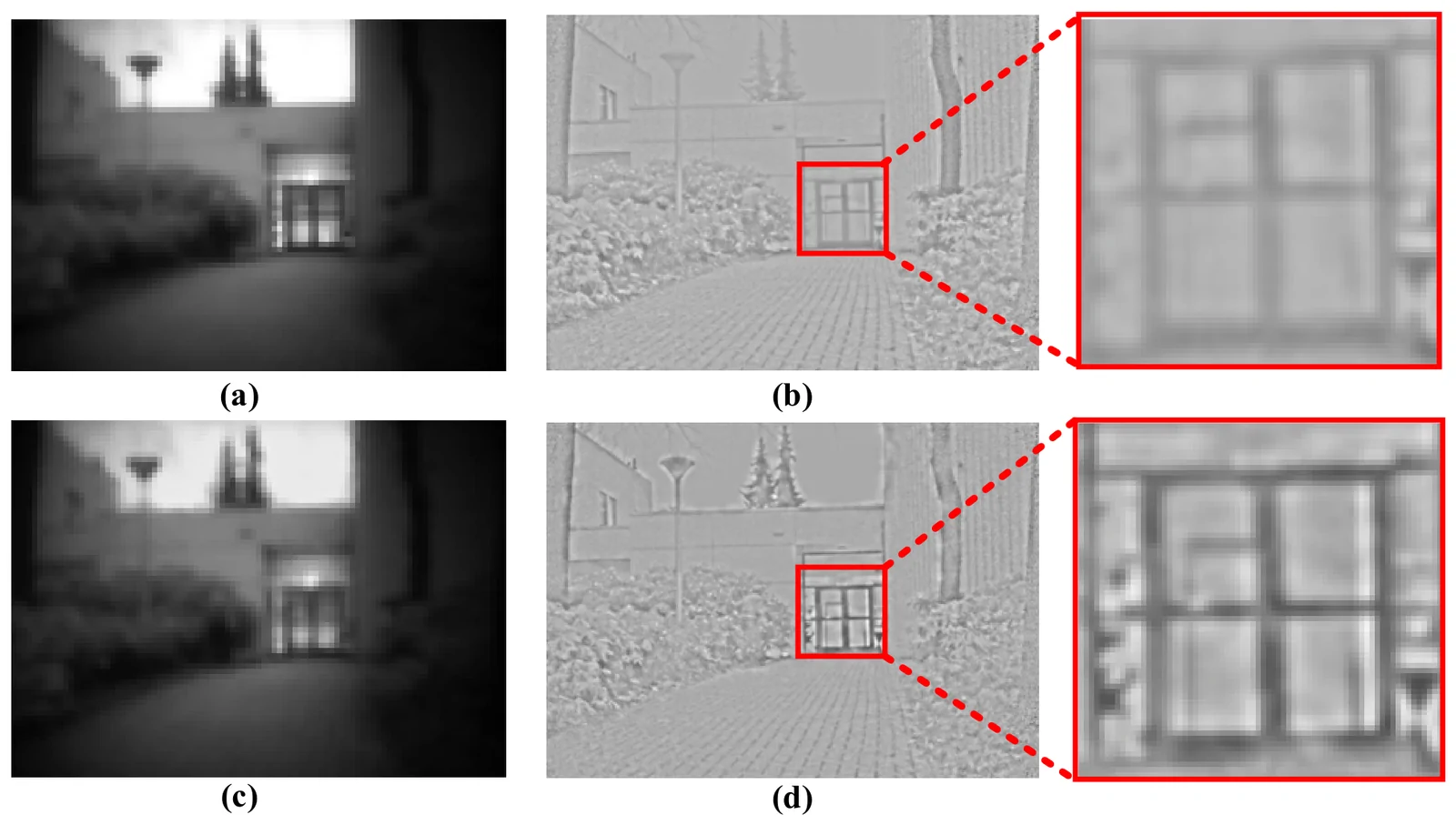
Decomposition results of guided filtering and deep guided filtering. (**a**) Base layer of guided filtering. (**b**) Base layer of deep guided filtering. (**c**) Detail layer of guided filtering. (**d**) Detail layer of deep guided filtering.

In this formulation, ${\left\|\cdot \right\|}_{1}$ denotes the L1 norm. At this juncture, the guidance image extraction procedure of the proposed deep guided filtering is completed. To substantiate the efficacy of deep guided filtering, Figure 3 presents a comparative visualization of the decomposition results obtained by conventional guided filtering and the proposed deep guided filtering, with both methods adopting identical parameter configurations—namely, a filtering window size of 6 and an edge-preserving degree (epsilon) of 0.01. As can be observed, the base layer produced by deep guided filtering (Figure 3b) exhibits superior smoothing of fine-grained textures compared with that generated by guided filtering (Figure 3a). Meanwhile, a closer inspection of the enlarged regions delineated by the red boxes in the detail layers reveals that deep guided filtering (Figure 3d) yields substantially richer and clearer fine-scale details than its conventional counterpart (Figure 3c). The above comparative analysis corroborates that the proposed deep guided filtering outperforms conventional guided filtering in terms of smoothing fine-scale details.

### 2.2. Multilevel Image Decomposition Model Based on Deep Guided Filtering and Gaussian Filtering

Section 2.2 corresponds to Part 1 of the fusion framework shown in Figure 1. Conventional multi-scale decomposition methods typically separate an image into high-frequency and low-frequency layers, yet they fail to further exploit information at different scales. This limitation prevents fusion algorithms from devising tailored fusion strategies that account for the distinct characteristics of infrared and visible images, potentially leading to issues such as information disarray, background blurring, and contrast degradation when fusing these two heterogeneous modalities. To address the aforementioned fusion challenges, this paper introduces a multilevel decomposition model based on deep guided filtering, as summarized in Algorithm 1.
**Algorithm 1:** Algorithm of Multilevel Image Decomposition Model**Require:** source image $I_{K}$, guided image $G_{K}^{4,*}$, l-th level deep guided filtered image $I_{d}^{\left(l\right)}$, l-th level Gaussian filtered image $I_{g}^{\left(l\right)}$, deep guided filtering DGF(·), Gaussian filtering Gaussian(·) **Ensure:** l-th level texture information $TD^{\left(l\right)}$, l-th level saliency information $SD^{\left(l\right)}$, base information $B_{K}$l=1: 1: $I_{d}^{\left(1\right)}=DGF(I_{K},G_{K}^{4,*},r_{d}^{\left(1\right)},\varepsilon_{d}^{\left(1\right)}),\;r_{d}^{\left(1\right)}=2\;\mathrm{and}\;\varepsilon_{d}^{\left(1\right)}=0.01$; 2: $I_{g}^{\left(1\right)}=Gaussian(I_{K},\sigma_{g}^{\left(1\right)}),\;\sigma_{g}^{\left(1\right)}=2$; 3: $TD^{\left(1\right)}=I_{K}-I_{d}^{\left(1\right)}$; 4: $SD^{\left(1\right)}=I_{d}^{\left(1\right)}-I_{g}^{\left(1\right)}$; for l=2:L 5: $I_{d}^{\left(l\right)}=DGF(I_{d}^{\left(l-1\right)},G_{K}^{4,*},r_{d}^{\left(l\right)},\varepsilon_{d}^{\left(l\right)}),\;r_{d}^{\left(l+1\right)}=2\cdot r_{d}^{\left(l\right)}\;\mathrm{and}\;\varepsilon_{d}^{\left(l+1\right)}=\varepsilon_{d}^{\left(l+1\right)}/2$; 6: $I_{g}^{\left(l\right)}=Gaussian(I_{g}^{\left(l-1\right)},\sigma_{g}^{\left(l\right)}),\;\sigma_{g}^{\left(l+1\right)}=2\cdot \sigma_{g}^{\left(l\right)}$; 7: $TD^{\left(l\right)}=I_{g}^{\left(l-1\right)}-I_{d}^{\left(l\right)}$; 8: $SD^{\left(l\right)}=I_{d}^{\left(l\right)}-I_{g}^{\left(l\right)}$; end 9: $B_{K}=I_{g}^{\left(L\right)}$; 10: **return:** $TD^{\left(l\right)}$, $SD^{\left(l\right)}$ and $B_{K}$;

As established in the preceding analysis, deep guided filtering is capable of effectively preserving image edges while removing high-frequency texture information. Exploiting this desirable property, we apply deep guided filtering to perform a two-scale decomposition on the input image $I_{K}$ (where the subscript $K\in \left\{Vis,IR\right\}$ denotes the visible and infrared modalities), yielding a high-frequency layer TD and a low-frequency layer $I_{d}$:

(5) $$TD=\;I_{K}-I_{d}$$

The high-frequency layer TD primarily encapsulates fine-scale textural details of the image. The low-frequency layer $I_{d}$, obtained via deep guided filtering, mainly comprises coarse-scale structural information, yet it also retains certain edge information preserved by the deep guided filtering operation. However, given that the fusion strategy for the low-frequency layer is primarily designed to integrate coarse-scale structural information, these useful edge cues would otherwise be discarded. To circumvent this loss, we consider further decomposing the low-frequency layer. Apart from edge-preserving filters, Gaussian filtering has also been widely adopted in image decomposition [31]. As a linear low-pass filter, Gaussian filtering eliminates not only textural information but also a portion of edge information. Consequently, the edge information that is retained by deep guided filtering but suppressed by its Gaussian counterpart can be computed as follows:

(6) $$SD=I_{d}-I_{g}$$

In this formulation, SD denotes the high-frequency layer containing edge information, and $I_{g}$ is a Gaussian-filtered image. Equation (5) enables the separation of edge features from the low-frequency layer. If the decomposition operations described in Equations (4) and (5) are iterated for l times, the proposed multilevel image decomposition model is consequently derived as follows:

(7) $$TD^{\left(l\right)}=\left\{\begin{array}{l} I_{K}-I_{d}^{\left(1\right)},\;\;l=1\\ I_{g}^{\left(l-1\right)}-I_{d}^{\left(l\right)},\;l=2,3,\ldots ,L \end{array}\right.$$

(8) $$SD^{\left(l\right)}=I_{d}^{\left(l\right)}-I_{g}^{\left(l\right)},\;\;\;l=1,2,\ldots ,L$$

(9) $$B_{K}=I_{g}^{\left(L\right)}$$

In Equations (7)–(9), l denotes the number of decomposition levels. $TD^{\left(l\right)}$ represents the texture information at the l-th level, $SD^{\left(l\right)}$ denotes the saliency information (strong edges) at the l-th level, and $B_{K}$ is the base information. $I_{d}^{\left(l\right)}$ stands for the l-th level deep guided filtered image, and $I_{g}^{\left(l\right)}$ refers to the l-th level Gaussian filtered image. The quantities $I_{d}^{\left(l\right)}$ and $I_{g}^{\left(l\right)}$ are obtained as follows:

(10) $$I_{d}^{\left(l\right)}=\left\{\begin{array}{l} DGF(I_{K},G_{K}^{4,*},r_{d}^{\left(l\right)},\varepsilon_{d}^{\left(l\right)}),\;\;l=1\\ DGF(I_{d}^{\left(l-1\right)},G_{K}^{4,*},r_{d}^{\left(l\right)},\varepsilon_{d}^{\left(l\right)}),\;l=2,3,\ldots ,L \end{array}\right.$$

(11) $$I_{g}^{\left(l\right)}=\left\{\begin{array}{l} Gaussian(I_{K},\sigma_{g}^{\left(l\right)}),\;\;l=1\\ Gaussian(I_{g}^{\left(l-1\right)},\sigma_{g}^{\left(l\right)}),\;l=2,3,\ldots ,L \end{array}\right.$$

In Equations (10) and (11), DGF denotes the deep guided filtering operation, with $r_{d}^{\left(l\right)}$ and $\varepsilon_{d}^{\left(l\right)}$ representing its filtering window size and edge-preserving degree, respectively. Gaussian(·) denotes the Gaussian filtering operation, and $\sigma_{g}^{\left(l\right)}$ is its standard deviation. Equations (7)–(11) collectively constitute the multilevel image decomposition model based on deep guided filtering and Gaussian filtering. The above decomposition scheme not only achieves effective separation of texture, saliency, and base information at different scales—thereby enabling scale-specific fusion of each sub-component—but also facilitates the selection and injection of salient information from infrared and visible images into the final fused result.

### 2.3. Fusion of Decomposed Sub-Information

The texture information, saliency information, and base information derived from the multilevel decomposition model constitute distinct types of information. Therefore, to endow the final fused image with both prominent infrared targets and clear visible backgrounds with fine textural details, we devise three tailored fusion strategies according to the respective characteristics of these components.

#### 2.3.1. Texture Information Fusion

Section 2.3.1 corresponds to Part 2 of the fusion framework shown in Figure 1. Since the first-scale decomposition layer contains the finest-scale detail information of the entire image, we select $TD^{\left(1\right)}$ and $SD^{\left(1\right)}$ at this level as the texture information layer. Compared with infrared imagery, visible images are endowed with richer textural details and are more consistent with human visual perception. These textural details, which belong to small-scale features, are most susceptible to illumination variations. Under low-illuminance conditions, the texture details in visible images generally suffer from poor clarity and low contrast. If conventional detail fusion methods—such as the absolute-value-maximization rule—are directly applied, the fused image may incorporate excessive texture information from the infrared modality (which aligns poorly with human vision), thereby leading to suboptimal fusion performance. To address this issue, we propose a texture information fusion strategy based on an enhancement function.

First, the visible texture information needs to be enhanced. To this end, the detail enhancement function (DEF) designed in this work is defined as follows:

(12) $$DEF=2-{\left(\frac{WSIL(T)-0.5}{0.5}\right)}^{2}$$

In Equation (12), WSIL(·) denotes the weighted sum of the improved Laplacian, and T represents the input texture image. The Laplacian operator, which is highly sensitive to the second-order derivatives of image intensity, responds rapidly to pixel value variations and is therefore widely employed to assess image sharpness [32]. Regions in which grayscale values undergo drastic changes—i.e., textural details—yield high Laplacian responses, whereas flat or slowly varying regions produce low responses. To capture richer textural details while simultaneously suppressing noise interference, WSIL(·) is defined as follows:

(13) $$WSIL(p,q)=\sum_{m=-r}^{r}\sum_{n=-r}^{r}W(m+r+1,n+r+1)\cdot IL(p+m,q+n)$$

In Equation (13), W is a (2r+1)×(2r+1) weighting matrix with radius r. For each element in W, its value is set to $2^{2r-d}$, where d is its four-neighborhood distance to the center. As an example, the 3 × 3 normalized version of W is:

(14) $$W=\frac{1}{16}\left[\begin{array}{ccc} 1 & 2 & 1\\ 2 & 4 & 2\\ 1 & 2 & 1 \end{array}\right]$$

IL(·) denotes improved Laplacian. Compared with the Laplacian, the improved Laplacian takes the effect of diagonal coefficients into account to make full use of neighboring information. The weights of two diagonal terms are set to $1/\sqrt{2}$ according to the Euclidean distance.

The expression for IL(·) is given as follows:

(15) $$\begin{array}{ll} IL(p,q)= & \left|2T(p,q)-T(p-1,q)-T(p+1,q)\right|\\ & +\left|2T(p,q)-T(p,q-1)-T(p,q+1)\right|\\ & +\frac{1}{\sqrt{2}}\left|2T(p,q)-T(p-1,q-1)-T(p+1,q+1)\right|\\ & +\frac{1}{\sqrt{2}}\left|2T(p,q)-T(p-1,q+1)-T(p+1,q-1)\right| \end{array}$$

As can be inferred from Equation (12), when the value of WSIL(T) approaches 1, it indicates that the corresponding visible texture detail exhibits higher clarity, and consequently the enhancement function is applied with a smaller intensity increment to prevent overexposure and distortion. Conversely, when WSIL(T) tends toward 0, the pixel is located in a smooth region of the visible image, where the enhancement function is assigned a relatively small value to mitigate noise amplification. The maximum enhancement factor of the function is 2, which is attained at WSIL(T)=0.5. The enhanced visible texture details, denoted as $TD_{EN}^{\left(1\right)}$ and $SD_{EN}^{\left(1\right)}$. $TD_{EN}^{\left(1\right)}$ is obtained via the following formulation:

(16) $$TD_{EN}^{\left(1\right)}=DEF_{T}\cdot TD_{Vis}^{\left(1\right)}$$

where $DEF_{T}$ denotes the enhancement function for $TD_{Vis}^{\left(1\right)}$, and $DEF_{S}$ denotes that for $SD_{Vis}^{\left(1\right)}$. Similarly, $SD_{EN}^{\left(1\right)}$ can be obtained. At this point, the visible texture details have been appropriately enhanced. In general, larger pixel values in the texture detail features indicate greater saliency. Therefore, the final fused texture information, denoted as $FuT_{1}$ and $FuS_{1}$. $FuT_{1}$ is obtained via the following formulation:

(17) $$FuT_{1}=\max(TD_{EN}^{\left(1\right)},TD_{IR}^{\left(1\right)})$$

Similarly, $FuS_{1}$ can be obtained. Through the above enhancement-function-based texture information fusion strategy, the clarity and contrast of local details in the fused image are effectively enhanced.

#### 2.3.2. Saliency Information Fusion

Section 2.3.2 corresponds to Part 3 of the fusion framework shown in Figure 1. In this paper, the detail features obtained from the second to the l-th decomposition levels are regarded as saliency information. Saliency information is dominated by edge features, which belong to high-contrast feature information. To ensure that the fused image preserves more prominent large-scale edge features, we propose a contrast-detection-based method for saliency information fusion. It has been found that the local standard deviation filter and the local range filter are both effective in sharpening edge features and extracting their contrast, yet their performance exhibits slight differences. The local standard deviation filter computes the standard deviation of pixel values within a local window around each pixel in the image and assigns this value to the corresponding pixel in the output image. This filter reflects the overall contrast of the local region: if the pixel values within the window are very close to each other (e.g., in a flat sky region), the standard deviation will be small; conversely, if the window contains edges (e.g., grassland), the pixel values fluctuate more substantially, yielding a larger standard deviation. Therefore, the local standard deviation filter is suitable for detecting the contrast of background edge features. The local standard deviation filter (LSDF) is defined as follows:

(18) $$LSDF(p,q)=\sqrt{\frac{1}{M\times N}\sum_{p=1}^{M}\sum_{q=1}^{N}{\left(S(p,q)-\bar{S}\right)}^{2}}$$

In Equation (18), S denotes the input saliency information (i.e., edges), $\bar{S}$ represents the mean value of S, and M and N indicate the dimensions of the local window, i.e., M×N.

The local range filter computes the pixel value range (maximum minus minimum) within a local window around each pixel in the image and assigns this range as the output value for the corresponding pixel position [33]. This filter reflects the “extreme contrast”—i.e., the maximum possible grayscale jump amplitude within the window: a larger range indicates pronounced brightness variations (e.g., at object–background boundaries), whereas a smaller range suggests smooth grayscale transitions. Consequently, the local range filter is well-suited for detecting the contrast of foreground (target) edge features. For the input saliency information (edges) S(p,q), with a window W of size M×N (typically M=N and odd-sized) centered at pixel (p,q), the local range filter (LRF) is defined as follows:

(19) $$LRF(p,q)=\underset{(p,q)\in W}{\max}S(p,q)-\underset{(p,q)\in W}{\min}S(p,q)$$

In Equation (19), $\underset{(p,q)\in W}{\max}S(p,q)$ refers to the maximum pixel value within the local window, and $\underset{(p,q)\in W}{\min}S(p,q)$ refers to the minimum pixel value within the local window.

Next, we first construct the single weight model using the local standard deviation filter and the local range filter:

(20) $$\begin{array}{l} TSW_{LSDFVis}^{\left(l\right)}=LSDF(TD_{Vis}^{\left(l\right)})/\left(LSDF(TD_{Vis}^{\left(l\right)})+LSDF(TD_{IR}^{\left(l\right)}\right))\\ TSW_{LRFVis}^{\left(l\right)}=LRF(TD_{Vis}^{\left(l\right)})/\left(LRF(TD_{Vis}^{\left(l\right)})+LRF(TD_{IR}^{\left(l\right)}\right))\\ SSW_{LSDFVis}^{\left(l\right)}=LSDF(SD_{Vis}^{\left(l\right)})/\left(LSDF(SD_{Vis}^{\left(l\right)})+LSDF(SD_{IR}^{\left(l\right)}\right))\\ SSW_{LRFVis}^{\left(l\right)}=LRF(SD_{Vis}^{\left(l\right)})/\left(LRF(SD_{Vis}^{\left(l\right)})+LRF(SD_{IR}^{\left(l\right)}\right)) \end{array}$$

In Equation (20), $TSW_{LSDFVis}^{\left(l\right)}$ and $TSW_{LRFVis}^{\left(l\right)}$ denote the single weight models constructed from $TD_{Vis}^{\left(l\right)}$ via the LSDF(·) and the LRF(·), respectively, while $SSW_{LSDFVis}^{\left(l\right)}$ and $SSW_{LRFVis}^{\left(l\right)}$ denote those constructed from $SD_{Vis}^{\left(l\right)}$ via the LSDF(·) and the LRF(·), respectively.

Finally, the fusion weights for saliency information are defined as follows:

(21) $$\begin{array}{l} TTW_{Vis}^{\left(l\right)}=\frac{1}{2}(TSW_{LSDFVis}^{\left(l\right)}+TSW_{LRFVis}^{\left(l\right)})\\ STW_{Vis}^{\left(l\right)}=\frac{1}{2}(STW_{LSDFVis}^{\left(l\right)}+STW_{LRFVis}^{\left(l\right)})\\ TTW_{IR}^{\left(l\right)}=1-TTW_{Vis}^{\left(l\right)}\\ STW_{IR}^{\left(l\right)}=1-STW_{Vis}^{\left(l\right)} \end{array}$$

In Equation (21), $TTW_{Vis}^{\left(l\right)}$ and $STW_{Vis}^{\left(l\right)}$ denote the saliency information fusion weights for $TD_{Vis}^{\left(l\right)}$ and $SD_{Vis}^{\left(l\right)}$, respectively, while $TTW_{IR}^{\left(l\right)}$ and $STW_{IR}^{\left(l\right)}$ denote those for $TD_{IR}^{\left(l\right)}$ and $SD_{IR}^{\left(l\right)}$, respectively.

The final fused saliency information, denoted as $FuT_{l}$ and $FuS_{l}$, is obtained via the following formulation:

(22) $$\begin{array}{l} FuT_{l}=TTW_{Vis}^{\left(l\right)}\cdot TD_{Vis}^{\left(l\right)}+TTW_{IR}^{\left(l\right)}\cdot TD_{IR}^{\left(l\right)}\\ FuS_{l}=STW_{Vis}^{\left(l\right)}\cdot SD_{Vis}^{\left(l\right)}+STW_{IR}^{\left(l\right)}\cdot SD_{IR}^{\left(l\right)} \end{array}$$

Through the above contrast-detection-based saliency information fusion strategy, the high-contrast edge features in the fused image are effectively preserved.

#### 2.3.3. Base Information Fusion

Section 2.3.4 corresponds to Parts 4 of the fusion framework shown in Figure 1. In this paper, the Gaussian-filtered image obtained at the l-th decomposition level is taken as the base information. This base information primarily contains the luminance information of the image, which belongs to coarse-scale structural information. Owing to adverse weather conditions or suboptimal shooting timing, the captured visible images often suffer from insufficient brightness. If a simple averaging operation is adopted to fuse the visible base information with its infrared counterpart, the resulting fused image would similarly exhibit excessive darkness and poor quality [34]. To improve the visual quality of the fused image, we propose a base information fusion strategy based on the intensity transformation function of infrared images and grayscale difference.

First, the intensity transformation function of infrared images is adopted to adjust the luminance of the visible base information:

(23) $$B_{EN}=B_{Vis}^{\gamma }$$

In Equation (23), $B_{Vis}$ and $B_{EN}$ denote the visible base information before and after enhancement, respectively, and γ is the stretching coefficient used to control the degree of luminance enhancement. In this work, γ is obtained by modifying the infrared base information $B_{IR}$ via a sigmoid function:

(24) $$\gamma =\frac{1}{\left(1+e^{-B_{IR}}\right)}$$

The infrared base information—whose grayscale values are normalized to [0, 1] via Gaussian filtering—is adopted as the stretching coefficient γ. This is equivalent to assigning a spatially adaptive enhancement weight to each pixel of the visible base information. In cold background regions of the infrared image (which often correspond to dark areas in the visible image), the infrared values are very small, producing a strong nonlinear brightening effect that can significantly bring out visible background details originally buried in darkness—such as grass and wall textures. In warm target regions, where the infrared values are moderate, the luminance of the visible base information is effectively boosted but not excessively. In hot target regions, where the infrared values are high and close to 1, almost no stretching occurs, and the original luminance of the visible base information is preserved. Furthermore, when the infrared value approaches 0, the enhanced value $B_{EN}$ tends to its maximum regardless of the value of $B_{Vis}$. Consequently, the primary role of incorporating the sigmoid function is to shift the histogram of the infrared base information toward the mean, thereby suppressing small pixel values and preventing overexposure. At this point, the luminance of the visible base information has been effectively improved.

Next, the fusion weights $W_{IR}^{B}$ and $W_{Vis}^{B}$ for the infrared and visible base information are constructed using the difference image between the two modalities:

(25) $$\begin{array}{l} W_{IR}^{B}=0.5+\frac{I_{IR}/255-I_{Vis}/255}{2}\\ W_{Vis}^{B}=1-W_{IR}^{B} \end{array}$$

The final fused base information FuB is obtained via the following formulation:

(26) $$FuB=W_{IR}^{B}\cdot B_{IR}+W_{EN}^{B}\cdot B_{EN}$$

The underlying rationale of the above fusion weights is as follows. In general, the grayscale values of targets in infrared images tend to be higher than those in visible images. Consequently, when $I_{IR}/255>I_{Vis}/255$, the weight $W_{IR}^{B}$ exceeds 0.5, and the fused base information incorporates more infrared luminance information to ensure that the targets remain prominent. Conversely, when $I_{IR}/255<I_{Vis}/255$, the weight $W_{IR}^{B}$ falls below 0.5, and the fused base information integrates more visible luminance information to enhance details that would otherwise be obscured by highlights or darkness.

#### 2.3.4. Fused Image Reconstruction

Section 2.3.4 corresponds to Parts 5 of the fusion framework shown in Figure 1. The final fused image is obtained by integrating the three decomposed sub-components, namely the texture information, saliency information, and base information:

(27) $$FuI=\sum_{l=1}^{4}\left(FuT_{l}+FuS_{l}\right)+FuB$$

## 3. Experimental Design and Result Analysis

To validate the effectiveness of the proposed method for infrared and visible image fusion under nighttime scenarios, we conduct experiments from four perspectives: subjective evaluation, objective evaluation, ablation studies, and parameter sensitivity analysis.

Datasets: The tests and analyses are primarily conducted on representative datasets, including TNO, RoadScene, LLVIP, and MSRS, for which the visible and infrared images have been properly registered. Among them, TNO and RoadScene are primarily used to evaluate visual performance in classical fusion scenarios, while LLVIP and MSRS are more suitable for assessing fusion quality in nighttime or complex road scenes.

Comparison Methods: We select ten representative image fusion methods as baselines for comparison, including the anisotropic diffusion and K-L transform-based fusion algorithm (ADF) [35], the convolutional sparse representation-based fusion method (CSR) [36], the latent low-rank representation-based fusion algorithm (LatLRR) [14], the two-scale decomposition and saliency detection-based fusion method (TSIF) [37], the cross bilateral filtering-based fusion algorithm (CBF) [38], the VGG19-based fusion algorithm (DLF) [18], the ResNet-based fusion method (ResNet) [30], the generative adversarial network-based fusion algorithm (GAN) [24], the Classification Saliency-Based Rule for Visible and Infrared Image Fusion (CSF) [39], A Semantic-Aware Real-Time Infrared and Visible Image Fusion Network (SeAFusion) [40], Cross-Domain Long-Range Learning for General Image Fusion via Swin Transformer (SwinFusion) [41] and the Progressive Infrared and Visible Image Fusion Network based on Illumination Aware (PIAFusion) [42]. The above methods cover different technical routes, including classical fusion methods (ADF, CSF, LatLRR, TSIF, CBF, DLF, ResNet, GAN, CSF), SOTA (State-of-the-Art) methods (SeAFusion, SwinFusion), and methods specifically designed for nighttime imaging scenarios (PIAFusion), thereby providing a comprehensive reflection of the current state-of-the-art in infrared and visible image fusion. All competing methods are implemented using the source codes provided by their original authors. All deep learning-based methods used the official pre-trained weights released by their authors from public repositories, and were evaluated on the exact same test dataset as our proposed method.

Objective Evaluation Metrics: In the field of image fusion, objectively assessing the performance of fusion algorithms is of critical importance. To this end, we adopt eight objective evaluation metrics to comprehensively evaluate the fused results from multiple aspects: average pixel intensity (API), standard deviation (SD), average gradient (AG), entropy (EN), spatial frequency (SF), total loss of information measure (LABF), perceptual saliency-based metric (PS), and visual information fidelity (VIF) [43]. Specifically, API represents the mean of pixel grayscale values in the image, reflecting the average brightness of the fused image. SD measures the deviation of each pixel from the mean pixel value of the image or image patch; a larger SD generally indicates richer detail information (provided that noise interference is excluded). AG reflects the capability of detail representation, capturing subtle contrast and texture variations, and thus can characterize image sharpness—a higher AG implies richer image hierarchy and better clarity. EN, a concept borrowed from information theory, is employed in image quality assessment to quantify the amount of information contained in an image; a higher EN value indicates richer information content, suggesting higher image quality in the absence of noise. SF reveals the distribution of gradient information in the fused image; a higher SF corresponds to more abundant edge and texture details. LABF reflects the total loss of information; a lower LABF value indicates less information loss. PS measures the amount of perceptually salient information in the image, with larger values signifying more salient content. VIF is employed to evaluate the information fidelity of a target image; in the fusion context, it quantifies the amount of information from the reference image that can be retained in the fused result, with higher VIF values indicating better fusion quality.

Implementation Details: All experiments are conducted on a workstation equipped with an Intel Core i7 CPU at 2.5 GHz and 4 GB RAM, running MATLAB 2017a. The parameters of the proposed method are configured as follows: the maximum decomposition level is set to l=4; the initial filtering window size and edge-preserving degree of the DGF are set to $r_{d}^{\left(1\right)}=2$ and $\varepsilon_{d}^{\left(1\right)}=0.01$, respectively, and the initial standard deviation of the Gaussian is set to $\sigma_{g}^{\left(1\right)}=2$; at the l-th decomposition level, the filtering window size and edge-preserving degree of the DGF are set to $r_{d}^{\left(l+1\right)}=s\cdot r_{d}^{\left(l\right)}$ and $\varepsilon_{d}^{\left(l+1\right)}=\varepsilon_{d}^{\left(l+1\right)}/s$, respectively, and the standard deviation of the Gaussian is set to $\sigma_{g}^{\left(l+1\right)}=s\cdot \sigma_{g}^{\left(l\right)}$; the scale factor between adjacent levels, denoted as s, is set to 2.

### 3.1. Subjective Evaluation

**Figure 4:**
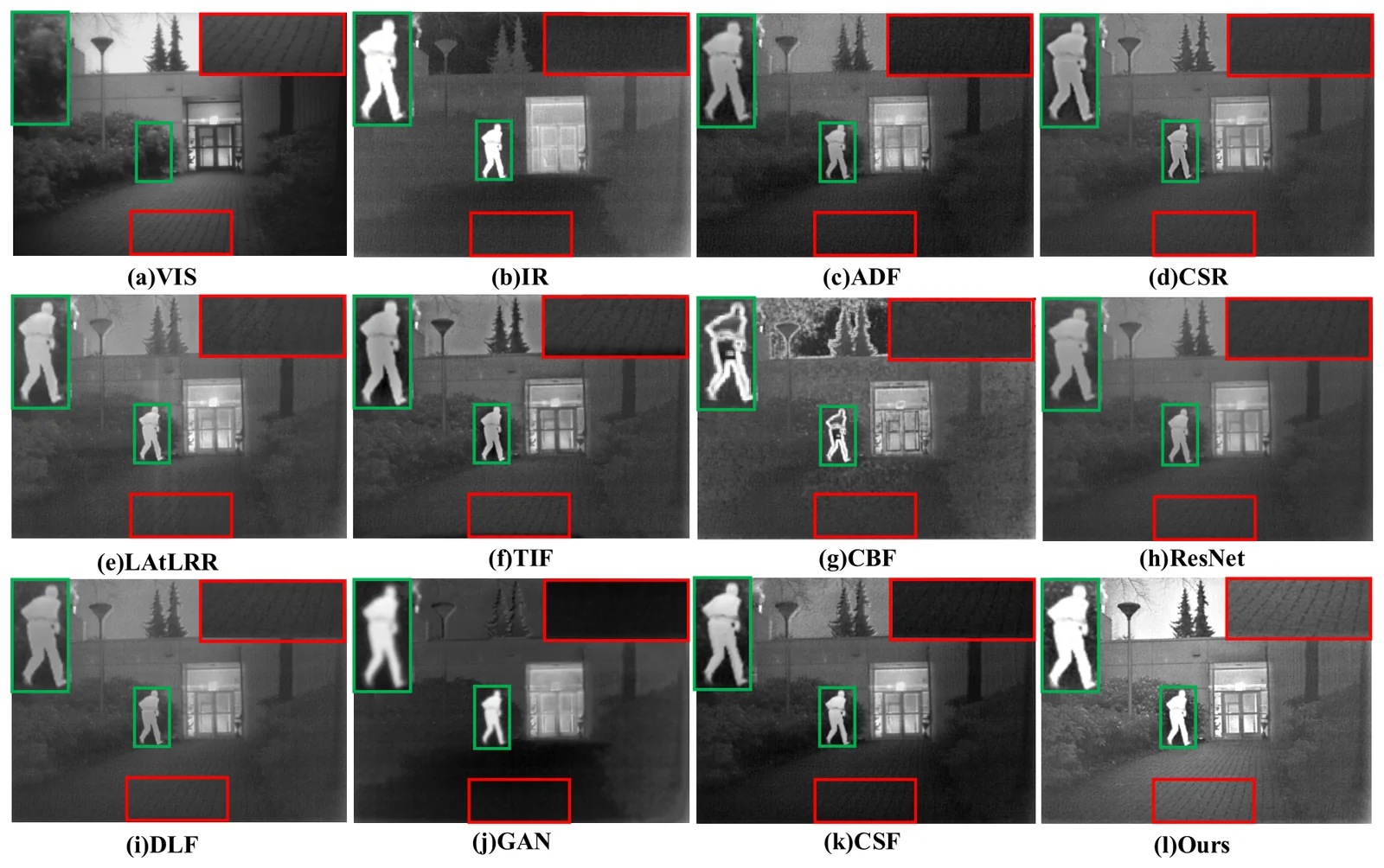
Comparison of different fusion methods on the image pair “Scene 1”.

**Figure 5:**
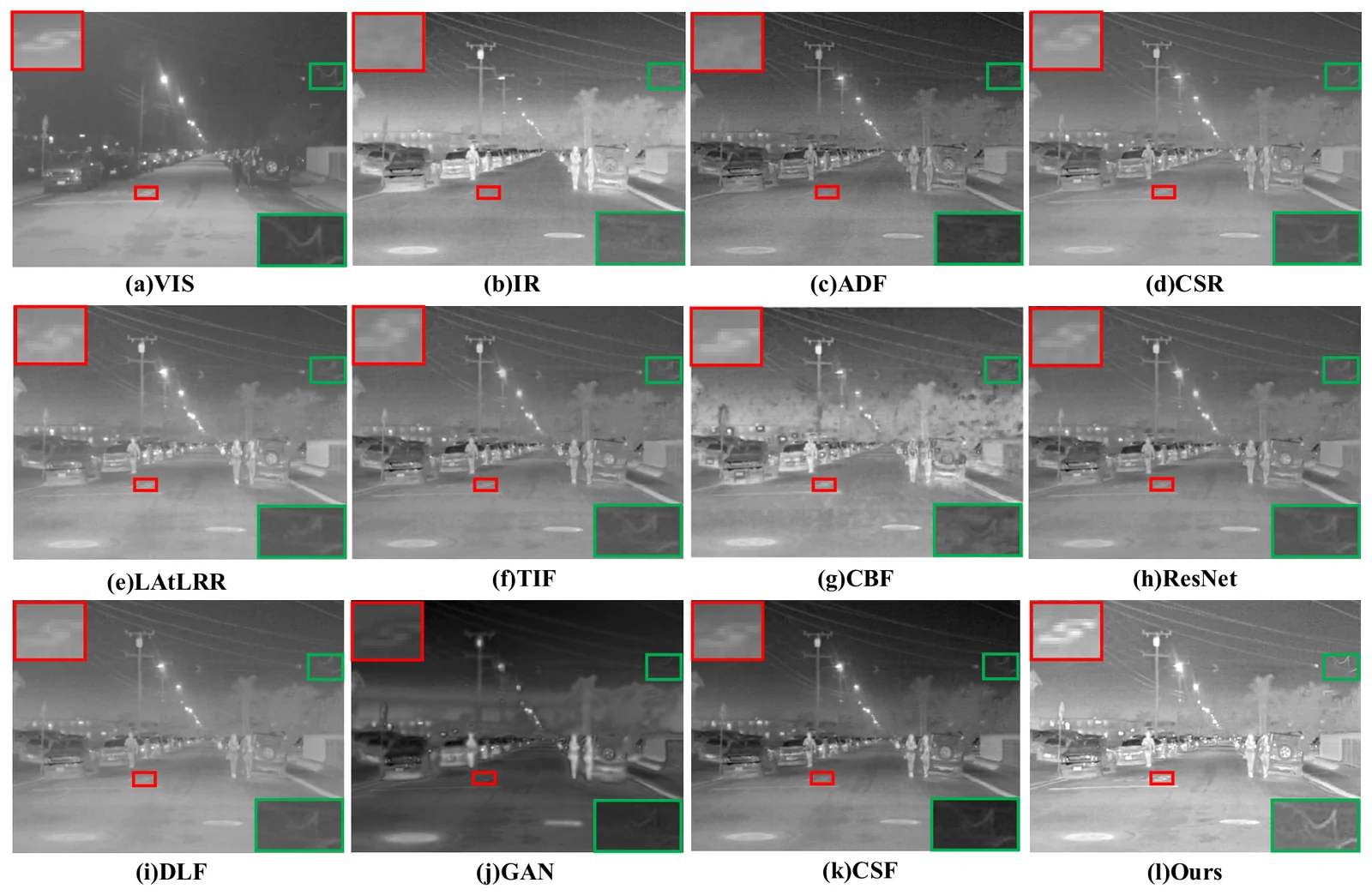
Comparison of different fusion methods on the image pair “Scene 2”.

**Figure 6:**
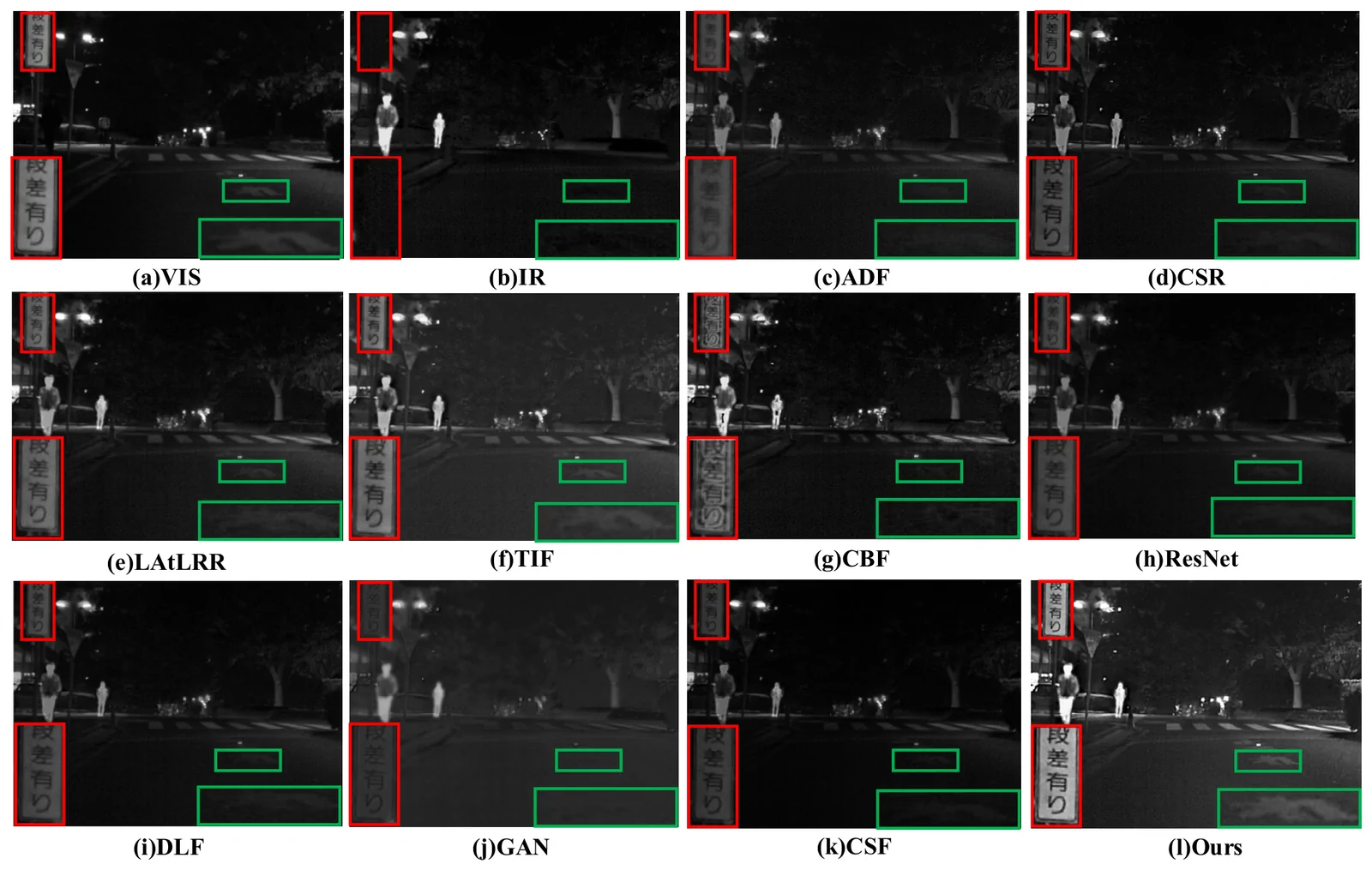
Comparison of different fusion methods on the image pair “Scene 3”.

**Figure 7:**
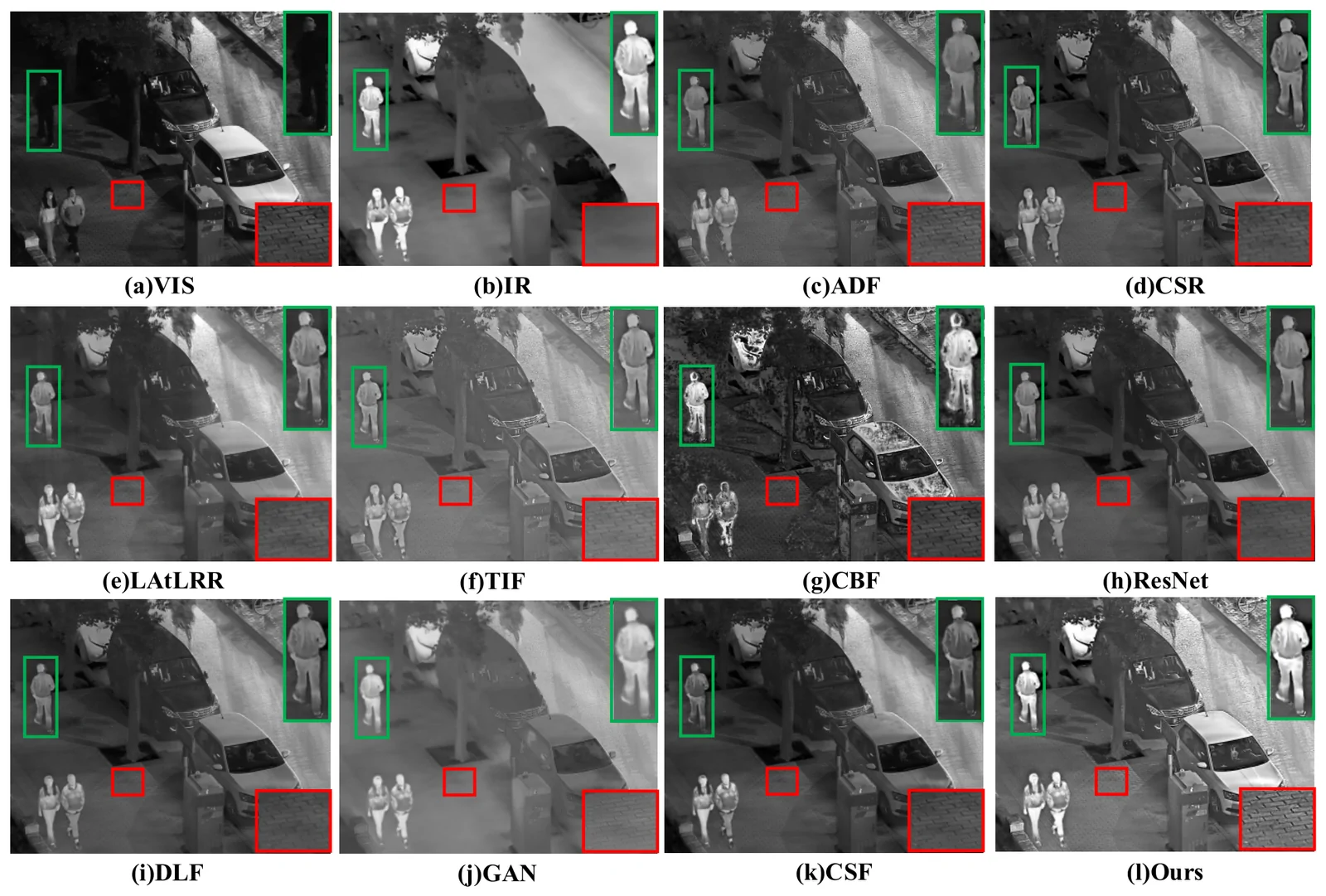
Comparison of different fusion methods on the image pair “Scene 4”.

For detailed comparative analysis, we select one representative image pair from each of the TNO, RoadScene, LLVIP, and MSRS datasets, denoted as “Scene 1,” “Scene 2,” “Scene 3,” and “Scene 4,” respectively. The fusion results obtained by different methods on these test images are presented in Figure 4, Figure 5, Figure 6 and Figure 7, respectively.

Figure 4 shows the fused images produced by each method on the source image pair “Scene 1.” As can be observed from Figure 4, the fusion results generated by ADF, GAN, and CSF suffer from insufficient brightness, leading to poor visibility of fine details—such as leaves and branches of trees and shrubs, as well as textures of pavement bricks. The results of DLF and ResNet exhibit relatively low sharpness in local details. LatLRR produces noticeable halo artifacts. CSR fails to capture sufficient saliency information from the infrared image, resulting in low contrast of infrared targets. The fusion images obtained by CBF and TSIF contain more extraneous information not present in the source images. In contrast, the proposed method successfully preserves more saliency and detail information from both the infrared and visible images. Notably, in the red and green boxes of Figure 4, compared with other algorithms, the proposed method achieves the best contrast for infrared thermal targets, with improved image brightness and the highest clarity and contrast of local details. These observations demonstrate that, from a subjective evaluation perspective, fused images generated by proposed method possess superior visual quality.

From the results presented in Figure 5, it can be observed that the fusion images generated by the nine competing algorithms are not sufficiently clear and tend to become blurry in certain detailed texture regions (e.g., the green and red boxes in Figure 5). Compared with the other algorithms, the proposed method achieves superior fusion performance from a subjective evaluation perspective, delivering favorable results in terms of both detail preservation and saliency feature retention. Notably, in terms of detail preservation, the proposed method performs the most aggressive enhancement of fine details, resulting in significantly clearer details in the fused image—as exemplified by the text within the red box and the power lines within the green box.

In the green-box region of Figure 6, none of the competing fusion algorithms are able to preserve the visible characteristics of the road text markings—each introduces certain alterations—whereas the proposed method successfully maintains this information as it appears in the visible image. This indicates that the other methods over-incorporate infrared information into the background regions. In the red-box area (billboard), the fused image generated by the proposed method exhibits significantly clearer details compared with those produced by the other algorithms.

Figure 7 presents subjective evaluation results analogous to those in Figure 4, Figure 5 and Figure 6. This outcome is mainly because the comparison methods are designed for inputs under clear or near-ideal conditions, while overlooking the impact of illumination on the details of visible images. Consequently, their fused images in nighttime scenes often exhibit unclear or invisible details and inconspicuous targets. Applying these fusion algorithms to downstream vision tasks would also lead to performance degradation. In contrast, the proposed algorithm first analyzes the susceptibility of the visible modality to low illumination, noise, and blur in nighttime infrared and visible image fusion, and builds the fusion framework on this basis, thereby achieving favorable fusion performance under low-light conditions.

**Figure 8:**
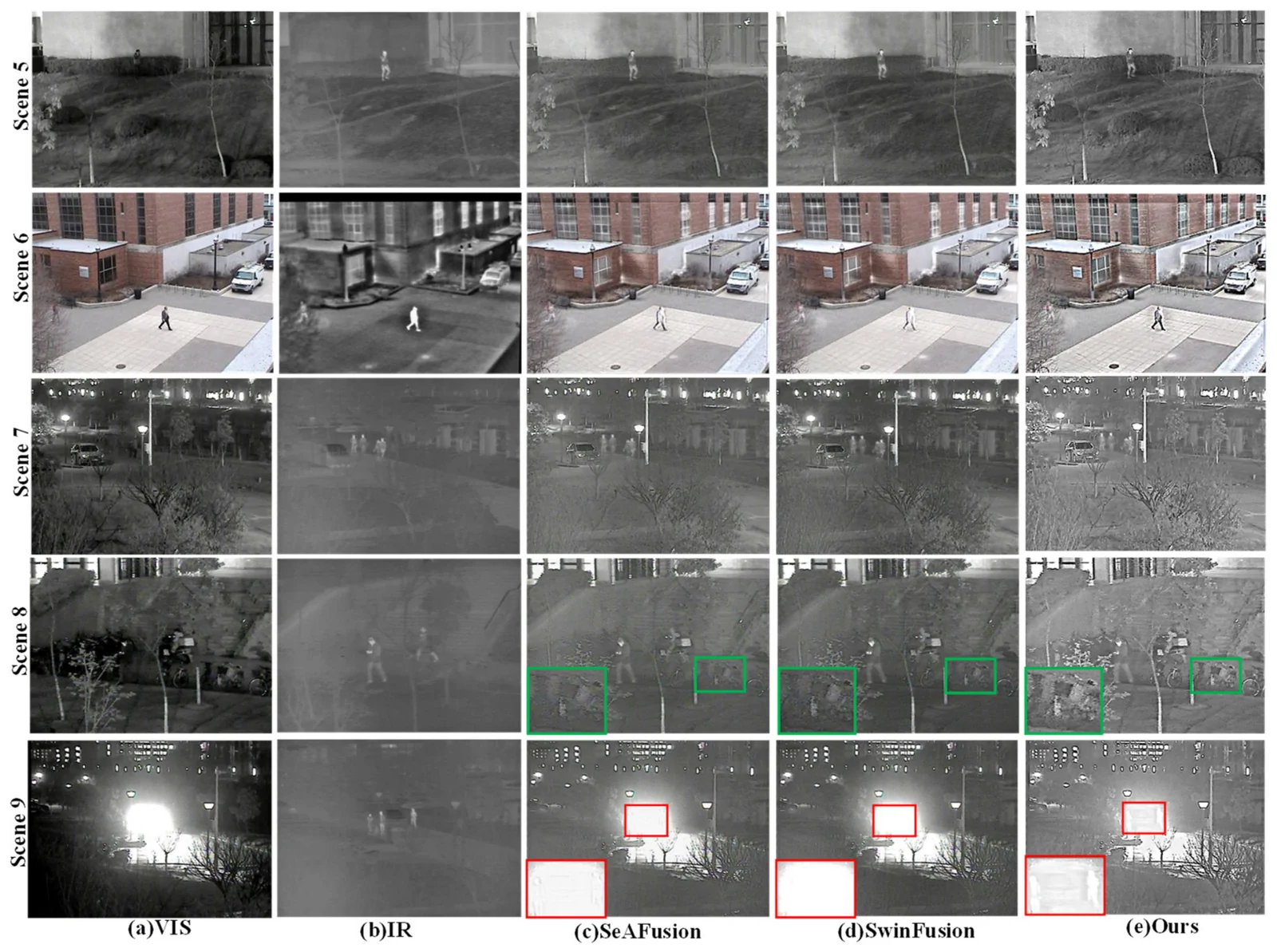
Comparison of different fusion methods on the image pair “Scene 5–9”.

In addition to the classical image fusion methods discussed above, the proposed method is also compared with several state-of-the-art (SOTA) fusion approaches. The fusion results of different methods across various scenarios are presented in Figure 8. As shown in Figure 8, SeAFusion, which cascades a semantic segmentation module and feeds high-level semantic information back to the fusion module via a semantic loss, achieves favorable performance in terms of scene brightness and infrared target saliency. However, its semantic guidance pathway comes at the expense of partially sacrificing gradient fidelity, leading to insufficient preservation of texture details (see the zoomed-in local patch in Scenario 8). SwinFusion employs the hierarchical window self-attention mechanism of Swin Transformer to capture global contextual information, thereby exhibiting certain detail preservation and structural consistency under low-light degradation conditions in visible images. Nevertheless, its pixel-maximum-based fusion loss tends to select high-intensity pixels from the source images, and the network is overly biased toward thermal radiation information from the infrared modality during optimization, resulting in overall over-brightness, low contrast, and unclear target contours in the fused results (see the zoomed-in local patch in Scenario 9). In contrast, benefiting from deep guided filtering multilevel decomposition and specialized layer-wise fusion rules (texture enhancement, local contrast weights, and intensity transformation), the proposed method maintains detail clarity and target contrast even under degraded scenarios such as intense illumination and low-light, demonstrating that it is more suitable for nighttime fusion tasks.

**Figure 9:**
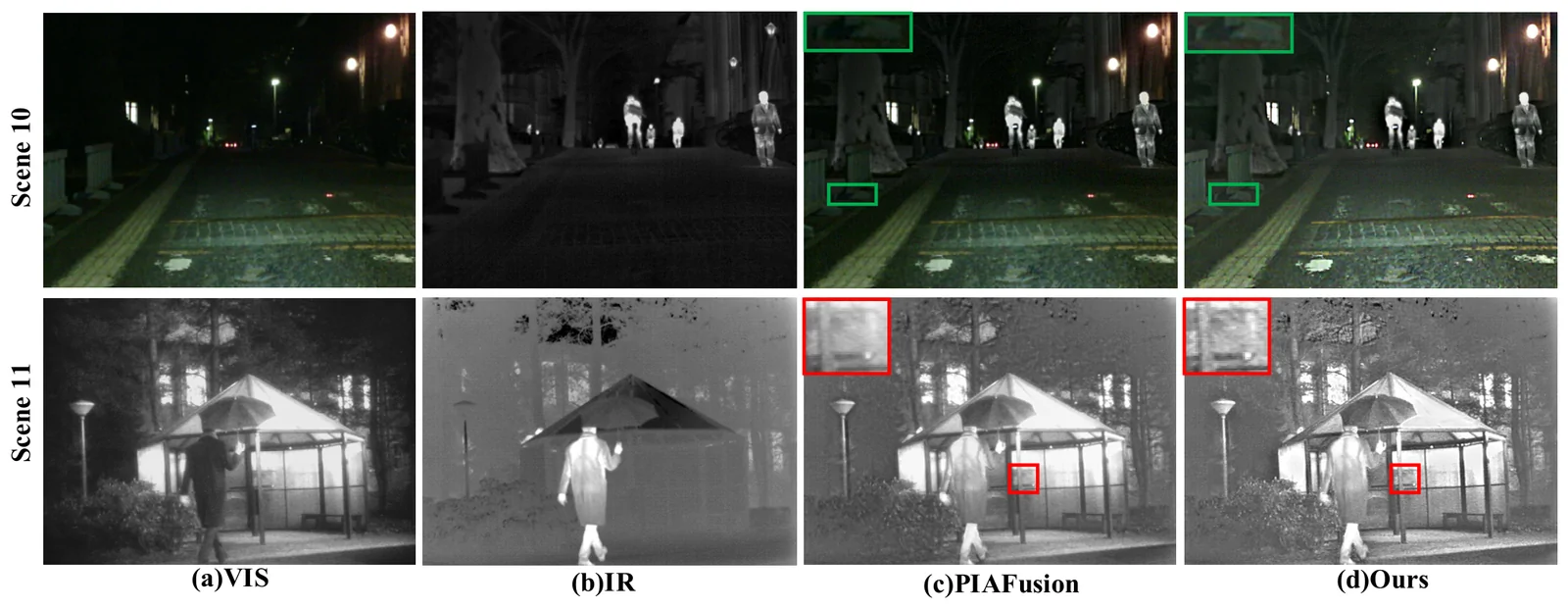
Comparison of fusion performance between PIAFusion method and proposed method.

To further validate the effectiveness of the proposed method in nighttime vision applications, we conduct a comparative experiment with PIAFusion—a fusion method specifically designed for nighttime imaging scenarios. As can be observed from Figure 9, the PIAFusion method achieves relatively high thermal infrared target contrast and preserves rich background details. However, its overall luminance enhancement remains limited, resulting in insufficient visibility of certain details in dark regions, such as the road guardrails in the green box. Moreover, in the green-box region of Figure 9, PIAFusion suffers from relatively poor local contrast.

This limitation arises because PIAFusion derives its weights from the global pixel statistics of the entire image. Although such a design can accommodate image-level illumination variations, it overlooks the spatial inconsistency of pixel distributions within local regions. When overexposed, underexposed, and normally illuminated areas coexist in the same image, the effectiveness of a globally uniform strategy is inevitably constrained. By contrast, the proposed method designs distinct adaptive fusion strategies according to the characteristics of different image scales, thereby enhancing the overall visual quality of low-light nighttime fused images. Brightness, contrast, and clarity are improved; fine details are accentuated; and essential source content is preserved. The resulting fused images are natural and clear, facilitating visual interpretation and analysis.

### 3.2. Objective Evaluation

As mentioned above, we adopt eight objective evaluation metrics—namely API, SD, AG, EN, SF, PS, LABF, and VIF—to assess the fusion performance of all competing methods. The metric values for both the comparison algorithms and the proposed method on six fused image pairs are reported in Table 1, Table 2 and Table 3, with the optimal values highlighted in bold. Table 1 presents the quantitative comparison between the proposed method and fusion algorithms designed for normal imaging scenarios. It can be observed from Table 1 that the proposed method achieves nearly all optimal values, significantly outperforming conventional algorithms such as ADF, CSR, LatLRR, TSIF, and CBF. Even when compared with several deep learning-based methods, including ResNet, DLF, GAN, and CSF, the proposed method still maintains a distinct advantage. These results demonstrate that the fusion images generated by the proposed method are of superior quality to those produced by the competing algorithms.

Table 2 further reports an objective comparison with state-of-the-art methods. It can be observed that, apart from VIF, the proposed method outperforms the competitors by a considerable margin on the remaining seven metrics, thereby further substantiating its superior performance. In addition, to further verify the improvement of the proposed algorithm in nighttime fusion, Table 3 provides an objective comparison between PIAFusion and the proposed method. PIAFusion is a fusion approach specifically designed for nighttime scenes and also falls into the category of deep learning-based methods. As shown in Table 3, except for SD and VIF in Scenario 5 and LABF and VIF in Scenario 6, the proposed method achieves the optimal values on all other objective evaluations, indicating its pronounced advantages in brightness, detail richness and clarity, as well as the salient feature content of fused images. This further demonstrates the effectiveness of the proposed method for night vision applications.

To compare the computational efficiency among different fusion methods, all evaluated algorithms were run on the same computer within the MATLAB environment. Table 4 presents the execution times of the compared methods when processing 1024 × 1280 source images. As can be seen, the proposed method does not rank first in terms of runtime efficiency; nevertheless, this trade-off is acceptable in view of its superior visual quality in the fused results. In summary, the subjective and objective experimental comparisons confirm that the proposed method is an effective fusion approach for nighttime vision applications.

### 3.3. Ablation Study Analysis

**Figure 10:**
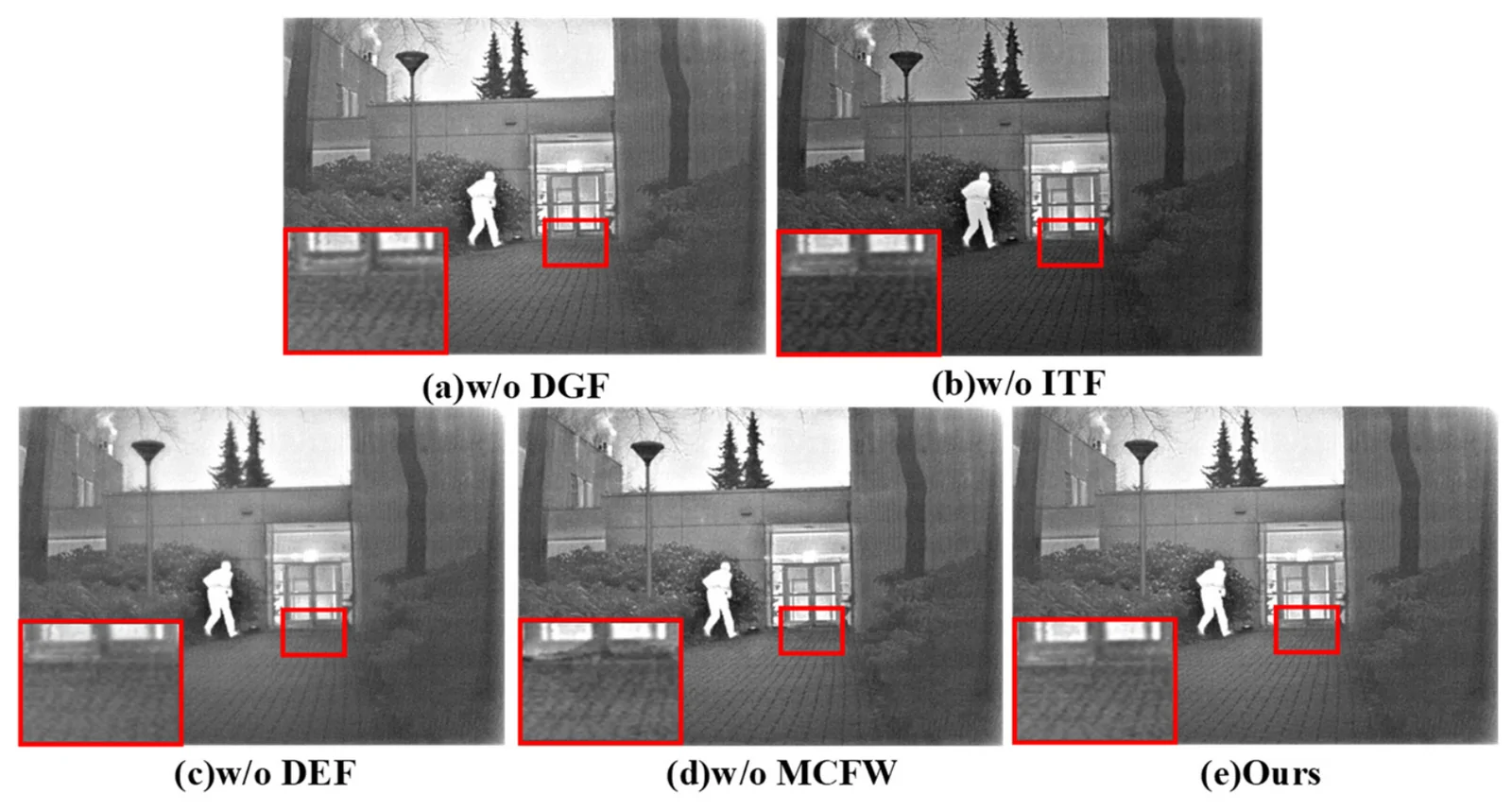
Visual ablation comparison of key fusion strategies of the proposed method.

In this experiment, we conduct ablation studies around four key components of the proposed method: deep guided filtering (DGF), intensity transformation function (ITF), detail enhancement function (DEF), and multi-contrast fusion weights (MCFW). Table 5 presents the quantitative results under different configurations, while Figure 10 further visualizes the fusion outcomes obtained by removing different fusion strategies.

As shown in Table 5, the full fusion algorithm achieves the best results on SD, EN, PS, and VIF, and ranks second-best on AG and SF. Although it does not attain the optimum on every metric, its subjective evaluation results are considerably superior to those of other strategy combinations (as illustrated in Figure 10), which is also of key importance for fusion tasks. Moreover, its objective values remain close to the best ones. This suggests that the fusion strategies employed in the full fusion algorithm are highly effective for this framework. Furthermore, when guided filtering is used in place of deep guided filtering, three optimal values (API, AG, SF) are obtained. This occurs because the adoption of guided filtering introduces more noise and artificially generated information into the fused image (as can be observed from the subjective evaluation results in Figure 10). Therefore, assessing the performance of a fusion algorithm solely by two or three metrics is far from sufficient; additional metrics should be adopted to evaluate it from multiple dimensions.

### 3.4. Parameter Discussion

For multiscale fusion methods, the decomposition level l directly affects the final fusion quality; therefore, its impact on the performance of the proposed method needs to be analyzed. Meanwhile, when determining the decomposition level, different adjacent scale coefficients s also influence the fusion performance, which likewise warrants further investigation.

**Figure 11:**
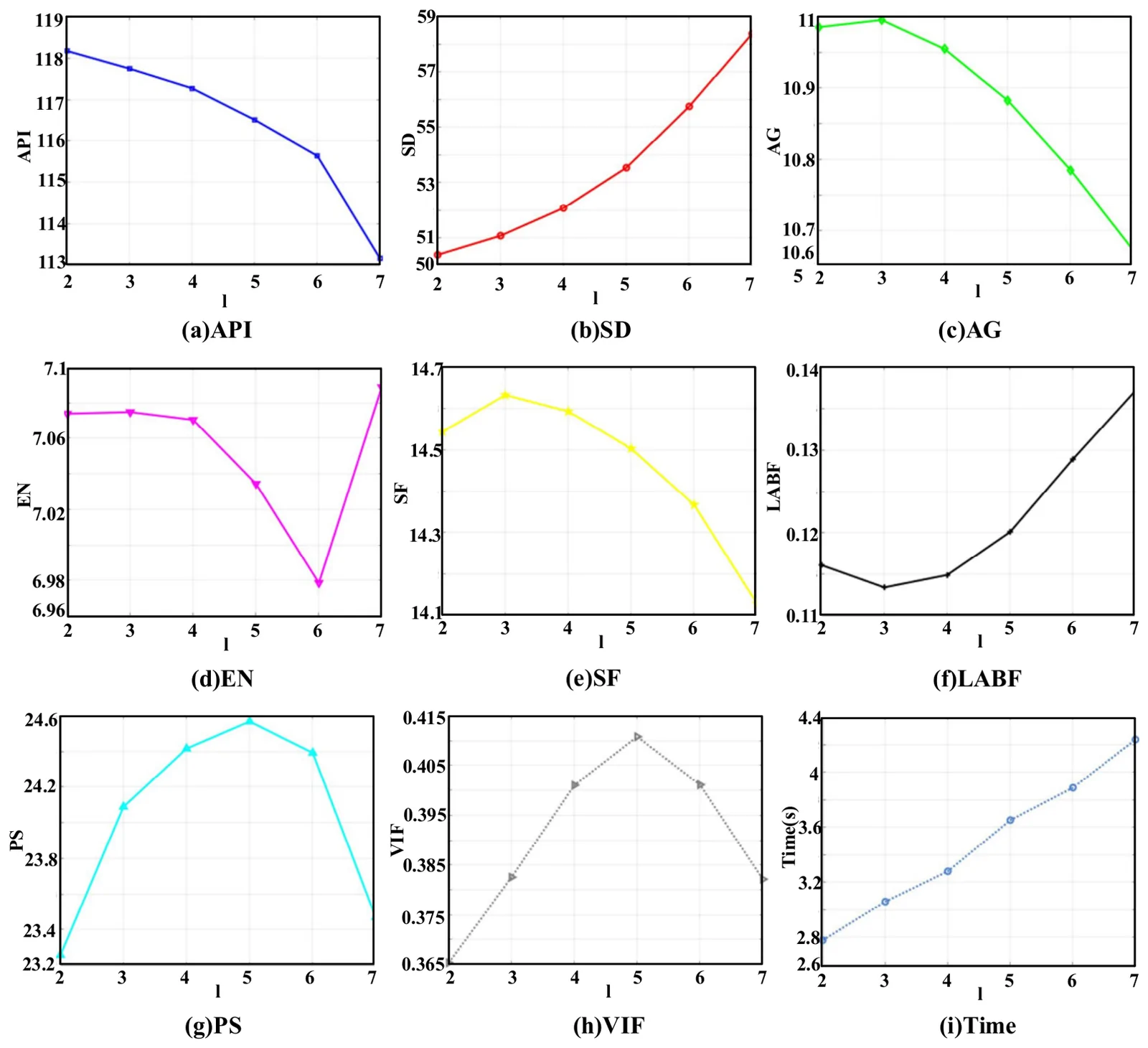
Performance comparison of the proposed method with different decomposition levels l.

Figure 11 presents the quantitative evaluation metrics of the fusion results under different decomposition level l. It can be observed from Figure 11 that as l increases, both API and AG exhibit a decreasing trend, while SD increases accordingly. The SF and LABF metrics reach their maximum values at l = 3, EN attains its minimum at l = 6, and both PS and VIF achieve their peak at l = 5. Figure 11i illustrates the impact of the decomposition level l on the runtime of the proposed method, revealing that the fusion time increases progressively with larger l, indicating that an excessively high decomposition level is clearly inadvisable. Conversely, when the decomposition level is too low, the advantages of multiscale decomposition cannot be fully exploited, making it difficult to obtain satisfactory fusion performance. Therefore, a decomposition level of l = 4 is considered the most appropriate, as it yields relatively optimal fusion performance while minimizing computational cost.

**Figure 12:**
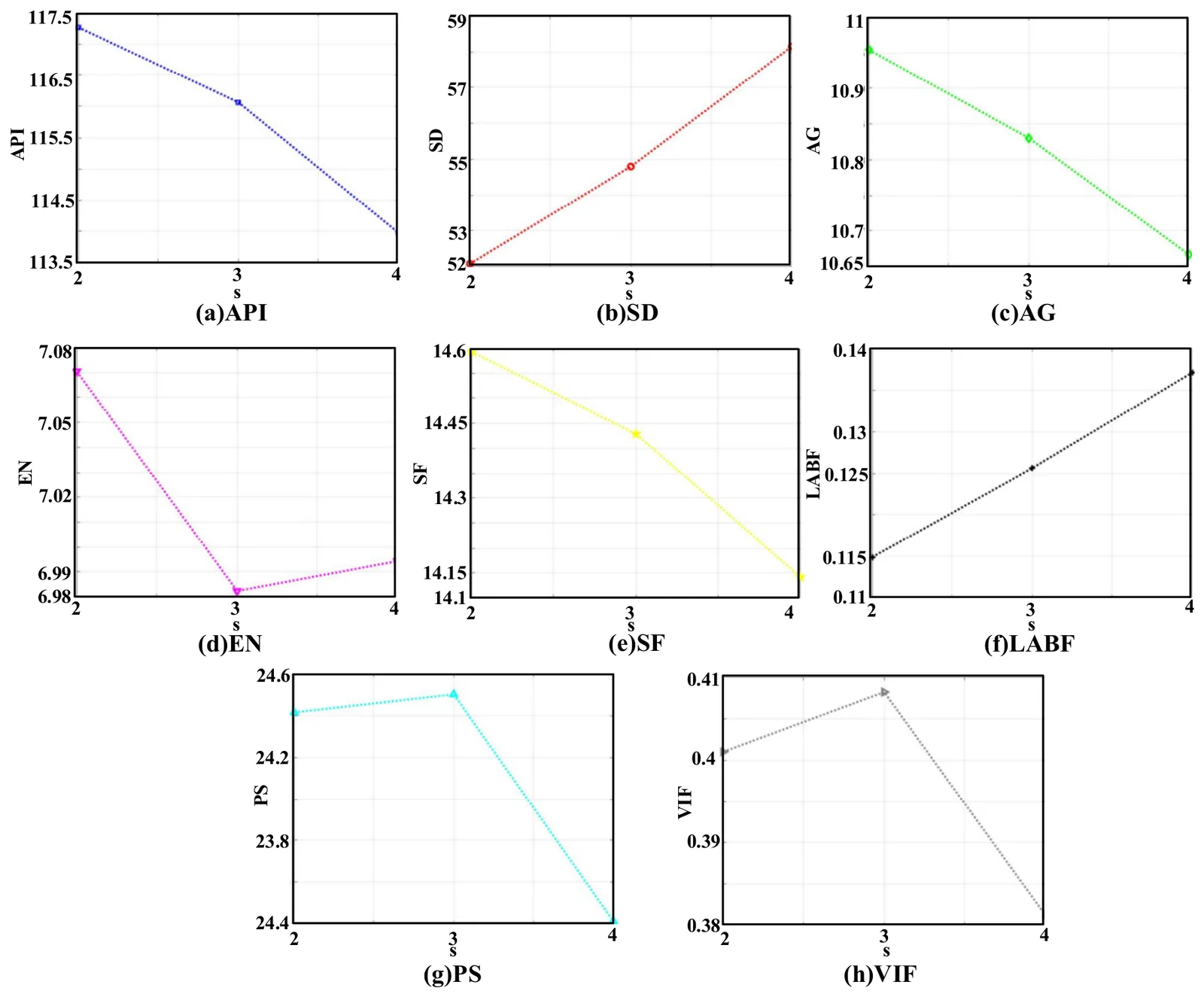
Performance comparison of proposed method with different adjacent scale coefficients s.

Figure 12 presents the quantitative evaluation metrics of the fusion performance under different adjacent scale coefficients s. As can be observed from Figure 12, the values of API, EN, AG, SF, and LABF achieve their optimal results at s = 2, while PS and VIF reach their best values at s = 3, with the latter two metrics remaining nearly identical for s = 2 and s = 3. SD attains its maximum at s = 4. From the above analysis, it can be concluded that the majority of the evaluation metrics yield the best results when s = 2.

In summary, the proposed method requires only a few parameters to be tuned and exhibits strong generalizability across different images.

## 4. Conclusions

This paper addresses the problem of poor detail clarity and visibility in infrared and visible image fusion under nighttime scenarios, and proposes a fusion method based on deep guided filtering and detail recovery. By introducing key strategies including a deep guided filtering-based multilevel decomposition model, the intensity transformation function, the detail enhancement function, and multi-contrast fusion weights, the proposed method achieves notable improvements in luminance, thermal target contrast, and detail sharpness of the fused images. In the experimental section, extensive ablation studies are conducted on the parameters and key fusion strategies, with their respective contributions analyzed in detail. The comparative experimental results demonstrate that the fused images generated by the proposed algorithm are more natural in visual appearance and secure favorable performance in terms of objective evaluation metrics. These findings collectively confirm that the proposed algorithm is capable of delivering superior fusion performance in nighttime fusion tasks. The limitation of the proposed method lies in that, although the enhancement-function-based texture information fusion strategy can improve the detail clarity of the fused image, it still confronts the dilemma between noise amplification and detail loss. A possible solution is to make the enhancement function noise-aware: rather than simply determining the gain based on brightness, the enhancement strength is dynamically adjusted according to the local signal-to-noise ratio (SNR) or noise density. This paper has conducted an in-depth study on nighttime visible and infrared image fusion technology and achieved certain results. However, due to the limitations of the authors’ expertise and research time, some ideas remain unrealized, and some issues have not been further explored. For example, in multiscale decomposition, fixed-parameter guided filtering is difficult to adapt to both flat and textured regions simultaneously; adaptively adjusting the filtering parameters using local variance or peak signal-to-noise ratio (PSNR) can be considered. Meanwhile, for real-time implementation, using the previous video frame as the guidance image for the current frame can be considered to reduce processing latency.

## Figures and Tables

**Table 1:** Results of eight evaluation metrics for the fusion algorithms on test image pairs. Bold values indicate the best results.

Imgs	Method	API	SD	AG	EN	SF	PS	LABF	VIF
	ADF	82.0353	31.6915	5.6025	6.6047	6.8282	10.3986	0.2944	0.3183
	CSR	82.1258	32.2584	5.0050	6.5928	6.9462	14.3331	0.1977	0.3299
	LatLRR	82.1258	35.7570	4.7563	6.7015	6.4058	14.4563	0.2656	0.3401
	TIF	90.4976	36.6092	6.3332	6.8424	8.7313	18.5998	0.1907	0.3394
Scene 1	CBF	85.8077	33.1930	7.8999	6.7770	11.0181	21.1696	0.1876	0.2163
	ResNet	82.3475	32.1839	6.5468	6.5468	5.3173	11.7565	0.3310	0.3310
	DLF	82.2529	31.6482	4.2115	6.5366	5.3991	11.7335	0.3490	0.3305
	GAN	57.6533	34.9732	2.9203	6.7229	4.3894	17.7149	0.6795	0.2445
	CSF	60.2591	42.3515	5.4621	6.9288	7.4522	17.0084	0.2623	0.3267
	OURS	**117.2720**	**52.0832**	**10.9544**	**7.0704**	**14.5928**	**24.4154**	**0.1200**	**0.4101**
	ADF	124.1051	30.7542	5.1561	6.7542	7.3827	12.0778	0.1879	0.3757
	CSR	124.1955	30.9790	4.3188	6.7460	7.0205	13.2703	0.1491	0.3897
	LatLRR	130.0924	32.4496	3.9539	6.8652	5.9933	12.9010	0.2656	0.3997
	TIF	124.3737	33.2756	5.2377	6.8998	8.3363	15.7582	0.1497	0.4025
Scene 2	CBF	131.0167	36.5730	6.4581	7.0683	9.2916	16.0355	0.1353	0.3253
	ResNet	124.1043	30.2706	4.6460	7.2182	9.3491	18.8142	0.2016	0.4383
	DLF	124.2683	30.2910	3.3846	6.6900	4.9817	10.9598	0.3245	0.3768
	GAN	64.4334	29.0553	3.0514	6.6847	5.1849	12.6294	0.5368	0.2937
	CSF	90.3513	**39.9564**	4.4535	**7.1339**	6.8263	13.8661	0.2233	0.3937
	OURS	**157.0973**	33.3302	**6.9351**	6.8923	**11.0846**	**18.6274**	**0.1200**	**0.4224**
	ADF	18.6047	17.9666	4.2580	5.3201	6.0733	11.8194	0.3119	0.3672
	CSR	18.7770	19.5381	4.0857	5.2930	7.1541	15.9762	0.1207	0.3468
	LatLRR	25.4291	25.1110	4.3701	5.7578	6.9854	18.8349	0.1183	0.3570
	TIF	19.3773	24.6485	4.7674	5.4773	8.5264	20.0043	0.1396	0.3020
Scene3	CBF	21.9522	26.5564	5.6553	5.5051	9.3357	21.8592	0.0958	**0.3983**
	ResNet	18.6894	18.2384	3.1331	5.2602	5.0259	13.2046	0.3099	0.3342
	DLF	18.6875	18.1591	3.1957	5.2552	5.2219	13.3383	0.2782	0.3354
	GAN	37.4677	15.0189	1.5539	4.9190	3.1186	11.5724	0.7588	0.1559
	CSF	11.3245	16.8526	2.2816	4.6798	4.6088	14.7153	0.4984	0.2682
	OURS	**45.2419**	**32.5091**	**7.1706**	**6.0762**	**12.8473**	**26.1996**	**0.0792**	0.2922
	ADF	80.2412	43.2331	6.7178	7.2194	11.9890	18.4749	0.1653	0.4091
	CSR	80.2929	44.6311	9.8320	7.2478	18.9433	19.7192	0.0862	0.3911
	LatLRR	89.6502	46.0177	4.8728	7.2363	10.1719	23.4712	0.2547	0.3903
	TIF	80.4518	47.2832	7.4172	7.3281	15.1323	26.8691	0.0913	0.3762
Scene 4	CBF	73.1601	48.6812	10.2250	7.2860	17.5188	28.1549	0.0523	0.4190
	ResNet	80.4260	43.9056	4.6460	7.2182	9.3491	18.8142	0.2016	0.4383
	DLF	80.5469	42.9543	5.7431	7.1899	11.0386	18.3083	0.2144	0.4053
	GAN	114.1874	33.4708	3.8347	6.8880	7.4273	14.6677	0.5425	0.2971
	CSF	76.9049	48.6385	5.0802	7.3315	9.3460	19.0722	0.1988	0.4366
	OURS	**115.0970**	**57.5082**	**13.7046**	**7.5045**	**27.7033**	**41.7984**	**0.0299**	**0.4495**

**Table 2:** Objective evaluation metrics of the SOTA method and the proposed method on the test images. Bold values indicate the best results.

Imgs	Methods	API	SD	AG	EN	SF	PS	LABF	VIF
	SeAFusion	108.9918	33.8858	8.2348	6.5207	14.8497	24.5732	0.1360	0.3688
5–9	SwinFusion	101.3012	37.3121	8.9721	6.6400	16.0776	24.0826	0.0993	**0.4086**
	OURS	**126.3596**	**38.2687**	**15.5967**	**6.8294**	**28.4200**	**34.3492**	**0.0350**	0.3722

**Table 3:** Objective evaluation metrics of the PIAFusion method and the proposed method on the test images. Bold values indicate the best results.

Imgs	Methods	API	SD	AG	EN	SF	PS	LABF	VIF
Scene 5	PIAF	117.4738	**46.8764**	8.6914	6.8091	11.7825	19.7740	0.1492	**0.4045**
	OURS	**129.5873**	45.3440	**14.4658**	**6.8775**	**19.9817**	**25.1071**	**0.0821**	0.3635
Scene 6	PIAF	36.6245	34.4482	5.2184	6.4967	9.0222	24.5256	**0.0690**	**0.5830**
	OURS	**57.8649**	**36.0719**	**6.7476**	**6.7670**	**12.0409**	**26.4589**	0.0697	0.4696

**Table 4:** A comparison of execution time of different methods (unit: second).

Methods	ADF	CSR	LatLRR	TIF	CBF	ResNet	DLF	OURS
Time	2.813	185.437	1086.639	0.131	67.081	8.395	22.812	13.326

**Table 5:** Ablation results of key fusion strategies of the proposed method. Bold values indicate the best results.

DGF	ITF	DEF	MCFW	API	SD	AG	EN	SF	PS	LABF	VIF
	✔	✔	✔	**117.420**	51.942	**11.203**	7.067	**14.870**	24.401	0.114	0.397
✔		✔	✔	81.387	41.023	10.916	6.999	14.274	22.503	**0.098**	0.366
✔	✔		✔	117.409	51.339	8.360	7.066	11.112	22.323	0.131	0.409
✔	✔	✔		117.274	52.075	10.951	7.069	14.587	24.411	0.115	0.401
✔	✔	✔	✔	117.272	**52.083**	10.954	**7.070**	14.593	**24.415**	0.119	**0.410**

## Data Availability

The original contributions presented in this study are included in the article. Further inquiries can be directed to the corresponding author.
